# Manipulating Spin States by Metal Axial Coordination of Active Sites for Generating Valuable CH_4_ in CO_2_ Reduction

**DOI:** 10.1002/advs.202517166

**Published:** 2025-11-06

**Authors:** Min Zhang, Qi Zhao, Yixuan Gao, Lirong Zheng, Jin Ouyang, Na Na

**Affiliations:** ^1^ Key Laboratory of Radiopharmaceuticals Ministry of Education College of Chemistry Beijing Normal University Beijing 100875 China; ^2^ State Environmental Protection Key Laboratory of All Material Fluxes in River Ecosystems College of Environmental Sciences and Engineering Peking University Beijing 100871 China; ^3^ Beijing Synchrotron Radiation Facility Institute of High Energy Physics Chinese Academy of Sciences Beijing 100049 China; ^4^ Department of Chemistry College of Arts and Sciences Beijing Normal University at Zhuhai Zhuhai 519087 China

**Keywords:** double‐solvent method, dual metal single‐atoms, metal axial coordination, metal spin state, orbital splitting

## Abstract

Dual‐atom catalysts (DACs) exhibit superior catalytic performance with more active sites and diverse electronic structures. However, electronic spin state as the crucial factor is rarely explicitly studied, and the in‐depth understanding of the electronic structures remains a great challenge. Herein, double‐solvent method is adopted to encapsulate the Ni and Mn ions into the channels of Mn‐based ZIF ‐8 (Ni→Mn DAC) and Ni‐based ZIF ‐8 (Mn→Ni DAC), respectively. The introduced metal exhibits axial coordination around the metal active site with the distinct coordination environments, indicating the diverse electronic structures. Experimental investigations and theoretical calculations demonstrate that the improved CO_2_ reduction (CO_2_RR) activity of Ni→Mn DAC derives from the axial coordinated Ni atom‐induced spin‐state transition of Mn 3*d* ^3^ from low‐spin (*d_xy_* ↑↓ *d_z_^2^* ↑) to high‐spin (*d_xy_* ↑ *d_xz_* ↑ *d_yz_* ↑). This is attributed to the splitting of Mn 3*d* orbital with specific C─Ni─N─Mn─N_3_ coordination structure, which makes Mn *d*‐band center push toward the Fermi level and further strengthens the adsorption of ^*^CO. Based on the accelerated reaction kinetics, the strong interaction between Mn active sites and ^*^CO leads to the rapidly completion eight‐electron process for generating CH_4_. This work provides an efficient spin‐manipulation strategy for accelerating the generation of valuable CH_4_.

## Graphical abstract

By encapsulating Ni atoms into Mn zeolitic imidazolate framework‐8, the spin‐state transition of Mn 3*d* ^3^ from low‐spin to high‐spin was facilitated upon Ni axial coordination via Mn 3d orbital splitting. This facilitates the electronic donation and induces the closing of Mn d‐band center toward Fermi level to strengthen *CO adsorption, thereby generating valuable CH_4_ in CO_2_ reduction reaction.

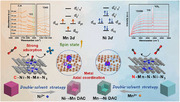

## 1 Introduction

Single‐atom catalysts (SACs) are known for definite single center with maximal atomic utilization, adjustable coordination environments, well‐defined active sites, and high catalytic selectivity.^[^ ^1^, ^2^, ^3^ ^]^ Many SACs have been employed for electrocatalytic CO_2_ reduction reaction (CO_2_RR) to generate high value‐added products.^[^ ^4^, ^5^ ^]^ However, SACs possess the insufficient ability to exceed the two‐electron process for generating the more valuable products in CO_2_ reduction. This is attributed to intrinsic drawbacks of SACs, which greatly impeded by the single top active sites for adsorption and the high activation barriers of transition states. To overcome the above problems, dual‐atom catalysts (DACs) with two adjacent metal active sites have gained tremendous interests in recent years, as an extension of SACs.^[^ ^6^, ^7^ ^]^ Compared with SACs, DACs have more active sites even bridge sites, different coordination environment, synergistic effect of metal‐metal.^[^ ^8^, ^9^ ^]^ It is beneficial to optimize the adsorption and desorption of complex intermediates via adjusting the *d*‐band center. Intriguing, the formation of dual‐atom sites can modify electronic structures to regulate the subsequent reactions for enhancing the catalytic performance.

**Scheme 1:**
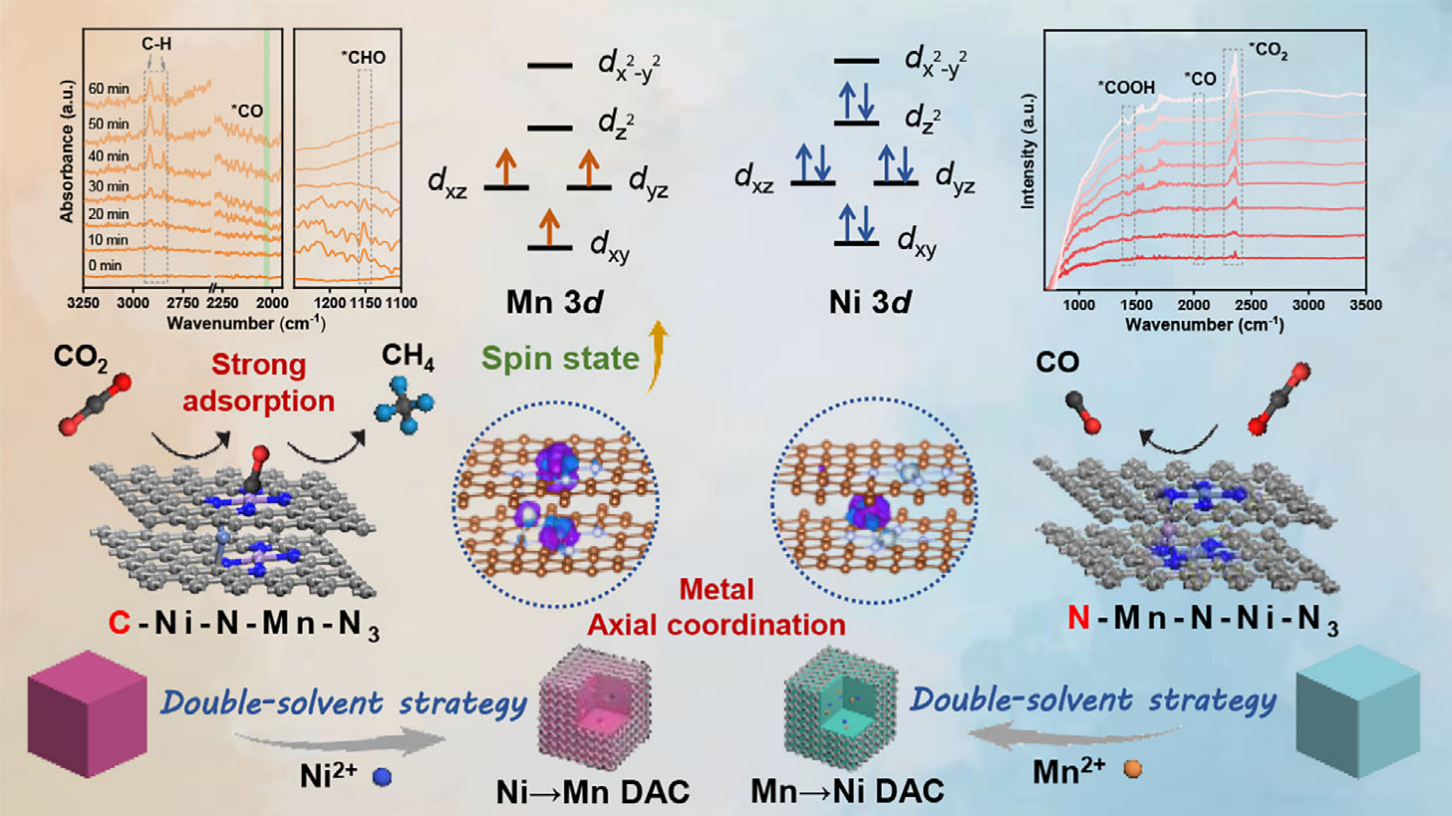
Schematic illustration of manipulating the spin state of active sites by metal axial coordination enables robust CO_2_ reduction with accelerating the generation of valuable CH_4_.

Recently, many dual‐atom sites have been constructed to modulate the electronic structure of catalytic sites to improve catalytic activity.^[^ ^10^, ^11^ ^]^ The intrinsic electronic and geometric characters of active sites are intensively relying on the surrounding microenvironment.^[^ ^12^, ^13^ ^]^ Therefore, the regulation of coordination environment for dual‐metal sites in DACs has been verified as an excellent strategy to engineer the electronic structure. Typically, the change in coordination environment leads to the adjustment of *d*‐band center, which can optimize the interaction between active sites and key intermediates during CO_2_RR.^[^ ^14^ ^]^ However, electron spin state, as the crucial inherent factor of electronic properties and the dominant element of chemical behavior for catalysts, lacking the detailed research. The reason is that manipulating the electron spin state of DACs to achieve superior reaction activity remains a formidable challenge with ambiguous understanding of explicit active sites in DACs and the precise synergistic effect. Furthermore, current studies predominantly focus on planar‐configuration DACs, demonstrating exclusive CO selectivity in CO_2_ electroreduction. This provides inadequate guidance for steering CH_4_ production, which greatly limits the application of DACs.^[^ ^15^, ^16^ ^]^ To address this limitation, Ni and Mn were selected as the ideal pair, primarily due to their highly tunable electronic structures and cost‐effectiveness as transition metals. It was hypothesized that constructing a Ni→Mn DAC with axial coordination, in which ligands bind along the axis perpendicular to the primary metal‐nitrogen plane. This would disrupt the symmetric electronic environment of planar configurations. The asymmetric structure is expected to modulate intermediate adsorption and enhance the performance towards CO_2_ reduction. Herein, we have encapsulated Ni and Mn ions into the channels of Mn‐based ZIF‐8 (Mn ZIF) and Ni‐based ZIF‐8 (Ni ZIF), obtaining Ni→Mn DAC and Mn→Ni DAC, respectively. Among them, the Mn→Ni DAC is only capable of producing CO, whereas the Ni→Mn DAC not only enables efficient generation of the target product CO but also demonstrates the potential for further conversion to valuable CH_4_ (**Scheme** **1**). This results in the metal dopant axially coordinates around the active metal on the ZIF‐8 surface, which can effectively regulate the spin state of active metal by orbital splitting. Intriguing, Ni→Mn DAC and Mn→Ni DAC possess different coordination environments for active site, which implies the distinct modulation of electronic structures. Specifically, the component of C‐Ni‐N‐Mn‐N_3_ within Ni→Mn SAC induces the Mn 3*d* orbital splitting, leading to the decreasing degenerate orbital energy level of *d_xz_* and *d_yz_*. Subsequently, the electronic rearrangement of Mn 3*d* ^3^ causes the spin state of Mn active site from low‐spin state (*d_xy_* ↑↓ *d_z_^2^* ↑) transform to high‐spin state (*d_xy_* ↑ *d_xz_* ↑ *d_yz_* ↑), accelerating the reaction kinetics of CO_2_ reduction. Moreover, the axially coordinated Ni atoms push the Mn *d*‐band center of Ni→Mn DAC toward the Fermi level, increasing the adsorption of ^*^CO key intermediate. Therefore, the strong adsorption between Mn active site and ^*^CO induces the rapid eight‐electron process of CH_4_ based on the accelerated dynamics. On the contrary, the N‐Mn‐N‐Ni‐N_3_ structure within Mn→Ni DAC exhibits ignored changes for the spin state of Ni 3*d* ^8^. Furthermore, the downward‐shifted Ni *d*‐band center represents weaker adsorption of the intermediates, which leads to the enhanced performance for generating single product CO.

## 2 Results and Discussion

### 2.1 Fabrication and Characterization of Metal Axial Coordinated Catalysts

**Figure 1:**
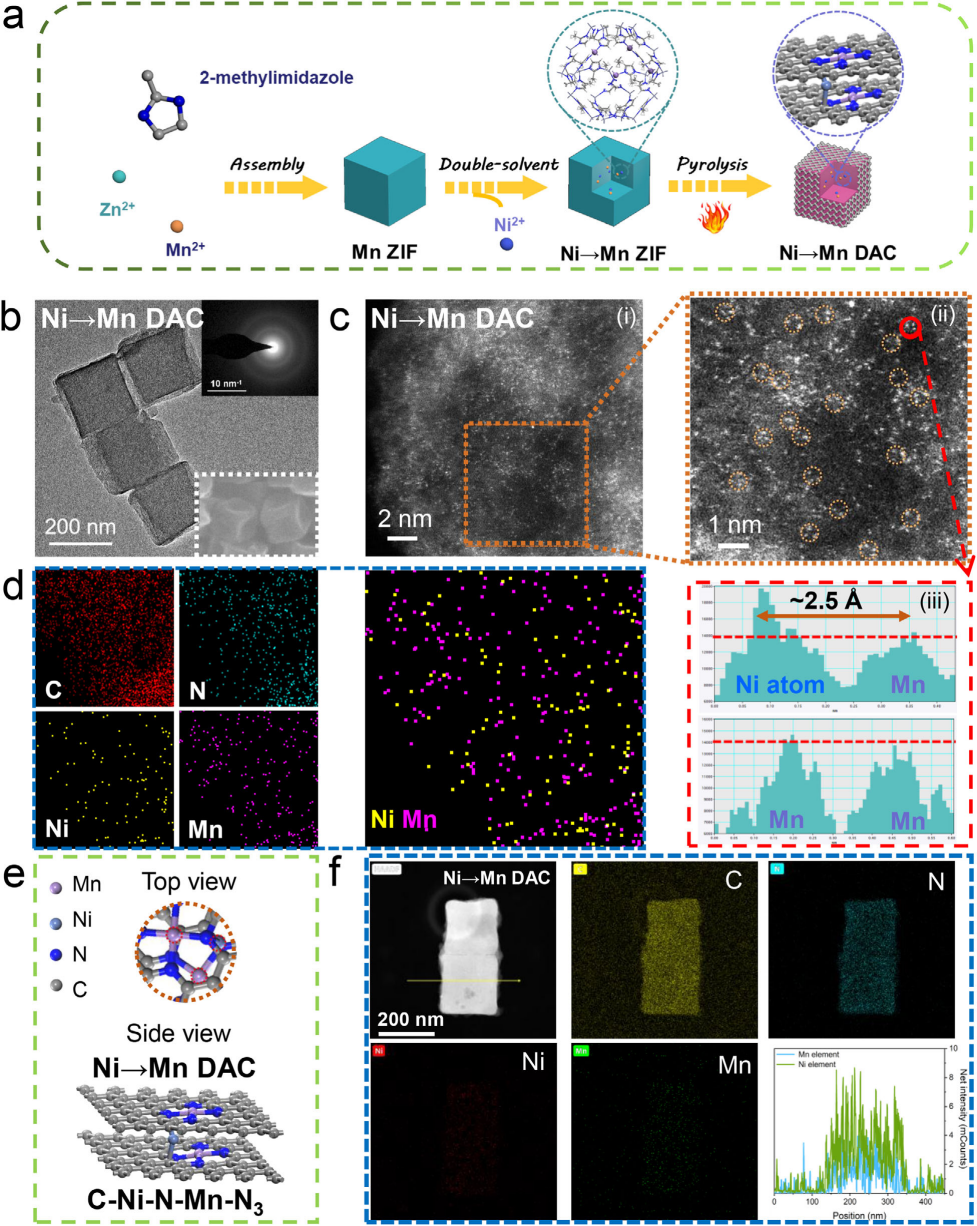
Synthesis characterization of Ni→Mn DAC. a) Schematic illustration of the synthetic process. b) TEM image (inset: the corresponding SAED and SEM images). c) i) HAADF‐STEM image, ii) corresponding partial enlargement, iii) intensity profiles obtained from the red highlighted region in image ii (red dotted line marks the same intensity). d) AC‐STEM‐EDS elemental mapping. e) The optimized model (top and side view). f) The corresponding EDS maps and elemental line profile.

The metal axial coordinated catalysts were synthesized through the double‐solvent method,^[^ ^17^ ^]^ which derived from porous metal‐based ZIF‐8 encapsulated heteronuclear metal within the channels. Briefly, the Mn ZIF was dispersed in *n*‐hexane uniformly, and an aqueous solution of Ni (II) ion was added dropwise. By virtue of the two‐solvent route combining polar and non‐polar media, the Ni (II) ion could be encapsulated within the ZIF‐8 cavities. Finally, thermal treatment resulted in volatilization of Zn nodes, and the Ni axial coordinated Mn catalysts (Ni→Mn DAC) with N‐doped carbon structure were successfully obtained (**Figure** **1**a). Notably, ZIF‐8 plays a crucial sacrificed template for providing N and C coordination atoms to stabilize Ni adjacent to Mn site. The internal Ni improves the catalytic activity via regulating the spin configuration of the Mn active site on the surface. As comparison, Mn→Ni DAC, Mn SAC, and Ni SAC catalysts were also fabricated through similar method as control materials.

The scanning electron microscopy (SEM) and X‐ray diffraction (XRD) reveal that Ni/Mn‐based ZIF‐8 remains the original structure after treatment with double solvent method (Figures S1a and S3a, Supporting Information). Compared to pristine ZIF‐8, all other metal‐incorporated ZIF‐8 catalysts maintained similar well‐defined crystallinity with no shift in XRD diffraction peaks, confirming the metal incorporation strategy does not compromise the structural integrity of ZIF frameworks.^[^ ^18^, ^19^ ^]^ After pyrolysis, catalysts still maintain the morphology of ZIF‐8 with slight size shrink (Figure 1b; Figure S1b, Supporting Information). The transmission electron microscopy (TEM) and high‐resolution TEM (HRTEM) images of single‐atom catalysts show a cubic structure without any metal nanoparticles. The selected area electron diffraction (SAED) image of single‐atom catalysts exhibit annular patterns characteristic of amorphous carbon (Figure 1b; Figure S1c, Supporting Information), which indicating that the atomically dispersion of metal.^[^ ^20^ ^]^ The XRD pattern of single‐atom catalysts feature similar carbon layer structure with two main peaks at ≈24° and 43°, which corresponding to (002) and (100) planes of carbon, respectively (Figure S3b, Supporting Information).^[^ ^14^ ^]^ In addition, the synthesized samples exhibit no characteristic diffractions ascribed to metallic crystals of Ni or Mn, which further verifies that the successful synthesis of single‐atoms catalysts. The Raman spectra in Figure S3c (Supporting Information) exhibit D and G band peaks at 1341 and 1578 cm^−1^, respectively. The synthesized samples possess similar rich disorientated graphene structures, as evidenced by the consistent intensity ratio of the D to G band (≈0.97). Moreover, Brunauer‐Emmett‐Teller (BET) was employed to evaluate the channel size of ZIF‐8. As shown in Figure S3d (Supporting Information), Mn SAC and Ni→Mn DAC display analogous pore size (≈0.86 nm), confirming metal could be incorporated in channel via double‐solvent method with negligible structural changes. Prior to pyrolysis, the precursor materials Mn ZIF and Ni→Mn ZIF show primary pore sizes of ≈0.52 nm (Figure S3e, Supporting Information). The observed pore size enlargement after pyrolysis, accompanied by a reduction in BET surface area, can be attributed to the collapse of the ZIF‐8 framework during the thermal treatment. This structural evolution further confirms that the introduced metal species remain atomically dispersed without forming aggregates, which would otherwise lead to pore blockage and a decrease in pore size.

To directly observe the atomic dispersion, the high‐angle annular dark‐field scanning TEM (HAADF‐STEM) was measured. In Figure 1c, many highly dispersed small bright dots (circled by the dotted lines) are throughout the C matrix, which is consistent with the SAED, HRTEM, and XRD results. Notably, neighboring tri‐metal sites were observed, and the isolated Ni and Mn atoms were identified by the intensity profiles difference of tri‐metal sites.^[^ ^21^, ^22^ ^]^ This demonstrates that Ni metal was successfully incorporated in Mn SAC by double‐solvent method. The aberration‐corrected electron microscopy elemental mapping overlay of Ni and Mn shows that both metals are independently distributed without aggregation (Figure 1d). Additionally, and the average distances between two adjacent Ni and Mn atoms were measured as ≈2.5 Å. The atomic distance is close to the metal bond length, in accordance with the optimized model in Figure 1e, suggesting the strong interaction between Ni and Mn atoms. The element distribution was elucidated by energy‐dispersive X‐ray spectroscopy (EDS) in Figure 1f and Figure S2 (Supporting Information), where Ni, Mn, and N were highly uniformly dispersed in the C matrix. And the result of elemental line profile also shows the presence of Ni metal, which is consistent with the above data. The loading contents of Ni and Mn in Ni→Mn DAC were determined to be 1.13 and 1.21 wt.% by inductively coupled plasma optical emission spectrometry (ICP‐OES) in Table S3 (Supporting Information).

### 2.2 Analysis of Local Chemical Configuration and Fine Structure

**Figure 2:**
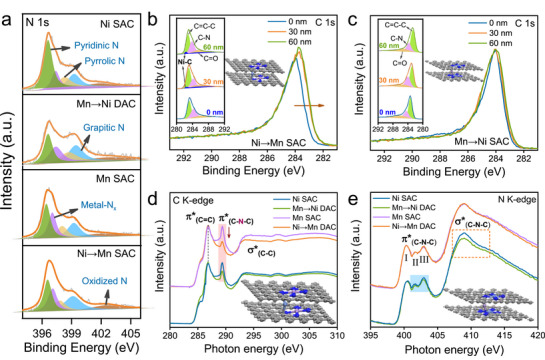
Local chemical configuration. High‐resolution XPS of a) N 1*s* for catalysts, C 1*s* spectra of b) Ni→Mn DAC and c) Mn→Ni DAC with Ar ion sputtering depths of 30 and 60 nm (inset: fitting curves and optimized models). d) C *K*‐edge and e) N *K*‐edge XANES spectra (inset: optimized models).

X‐ray photoelectron spectroscopy (XPS) was measured to characterize the surface and composition of catalysts. The high‐resolution N 1*s* spectra of all samples show five main peaks, which indicates the presence of pyridinic N, metal‐bonded N, pyrrolic N, graphitic N, and oxidized N (**Figure** **2**a).^[^ ^20^, ^23^ ^]^ The C 1*s* XPS spectra of Ni→Mn DAC show obvious peak broadening with the increase of Ar^+^ sputtering depth. The spectra can be deconvolved into four characteristic peaks, where the peak at ≈282.3 eV was attributed to Ni‐C coordination bond,^[^ ^22^, ^24^, ^25^ ^]^ as observed in the depths of 30 and 60 nm (Figure 2b). No similar signal is presented in Figure 2c, indicating that the existence of Mn‐N coordination bond within Mn→Ni DAC.

To gain insight into the coordination environment, synchrotron soft X‐ray absorption near‐edge spectroscopy (XANES) was performed. Compared with XPS, XANES is sensitive to local chemical configuration and partial electronic state. The C *K*‐edge spectra (Figure 2d) for all materials exhibit three main resonances (with peak centers of ≈286.8, 289.3, and 293.3 eV), which can be ascribed to the dipole transition of the 1*s* core electron of C into the π^*^_C═C_, π^*^_C─N─C,_ and σ^*^_C─C_ antibonding states, respectively.^[^ ^26^, ^27^, ^28^ ^]^ The preservation of the similar main features indicates that the axial coordinated metals induce tiny changes of framework. However, the variation of peak intensity of π^*^_C─N─C_ demonstrates the different coordination environment. Compared with Mn SAC, the significantly reduced intensity of Ni→Mn DAC suggests that the π^*^_C─N─C_ unfilled state was doped by exotic electrons. This is due to the axial Ni‐C coordination, leading to the destruction of the original C─N─C structure. On the contrary, Mn→Ni DAC exhibits subtle decrease in comparison to Ni SAC, which means that Mn‐N coordination bond alters the C─N─C moieties slightly. Furthermore, the N configurations were also selectively affected in the materials, which can be confirmed by the N *K*‐edge spectra. In Figure 2e, three typical peaks (I, II, and III) appear at ≈400.3, 401.6, and 402.8 eV, corresponding to the transition of N 1*s* (pyridinic N, pyrrolic N and graphitic N) into π^*^, respectively.^[^ ^29^, ^30^ ^]^ And a strong peak located at ≈408.8 eV is ascribed to electron transition from N 1*s* to σ^*^_C─N─C_ orbital. Remarkably, the signal of Mn→Ni DAC is weaker than that of Ni SAC, implying the presence of Mn‐N bonds destroys the C─N─C moieties. Moreover, peaks II and III also display coincident trend change (highlighted by the blue pattern). This is consistent with the results of C *K*‐edge spectra. The results provide solid evidences to prove that Ni‐C and Mn‐N moieties exist in the interior of Ni→Mn DAC and Mn→Ni DAC, respectively.

**Figure 3:**
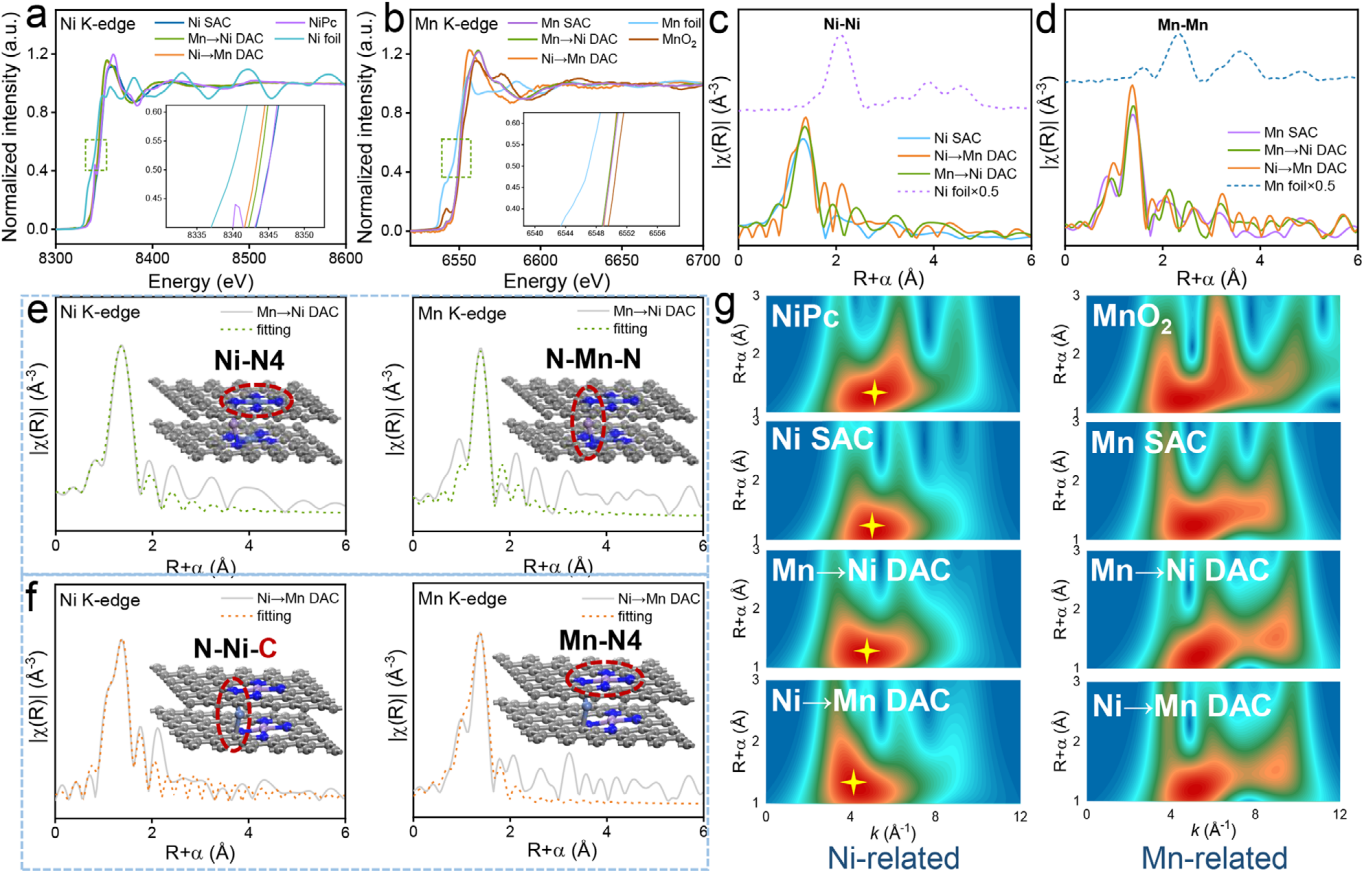
Atomic structure examinations. Normalized a) Ni *K*‐edge and b) Mn *K*‐edge XANES spectra (inset: enlarged spectra). FT *k* ^2^‐weighted c) Ni *K*‐edge and d) Mn *K*‐edge of the EXAFS spectra. The corresponding EXAFS fitting results for e) Mn→Ni DAC and f) Ni→Mn DAC (inset: optimized models). g) The corresponding WT EXAFS spectra.

To obtain detailed information on the valence states and coordination environments of the Ni and Mn atoms, X‐ray absorption spectroscopy (XAS) was performed. The X‐ray absorption near‐edge structure (XANES) of Ni SAC exhibits similar absorption edge to standard NiPc, indicating that the valence state of Ni specie is mainly +2 in **Figure** **3**a. Moreover, the absorption edge of Mn→Ni DAC and Ni→Mn DAC display negative shift, suggesting the lower oxidation states of Ni sites. On the contrary, the XANES of Mn *K*‐edge shows that the similar absorption edge of Mn‐related catalysts and MnO_2_ standard, implying that the Mn valence states are +4. The specific valence states of Ni and Mn states were calculated according to the absorption edges (Figure S4a,b, Supporting Information). The Fourier transforms (FT) *k* ^2^‐weighted extended X‐ray absorption fine structure (EXAFS) spectra of samples for both Ni and Mn *K*‐edge exhibit predominant metal‐N (metal = Ni, Mn) coordination at ≈1.4 Å (Figure 3c,d). No characteristic peaks for Ni‐Ni or Mn‐Mn coordination are detected in Ni SAC and Mn SAC, reconfirming the metal species are atomically dispersed.

To obtain more coordination information around the axial coordinated metal atoms, FT‐EXAFS fittings were performed (Figure 3e,f; Figures S4c,d and S5, Supporting Information). The coordination number (CN) of Ni SAC (Ni‐N path) and Mn SAC (Mn‐N path) are 3.7 and 3.8, respectively (Tables S1 and S2, Supporting Information). The best fitting results for metal‐N path in Mn→Ni DAC demonstrate that the CN of axial coordinated Mn and Ni atom are 1.8 and 3.8, respectively. This confirms the existence of the N‐Mn‐N‐Ni‐N_3_ configuration. Significantly, the axial coordinated Ni site of Ni→Mn DAC suitable fitting curves exhibit Ni‐C (CN = 1.1) and Ni‐N (CN = 1.2) scattering paths, which verified the C‐Ni‐N‐Mn‐N_3_ configuration and related to the results of XPS C1*s* spectra. To further distinguish coordination patterns for the axial coordinated metal atoms, wavelet transform (WT) analysis was carried out as an ideal complement for FT.^[^ ^31^ ^]^ As shown in Figure 3g, the intensity maximum at ≈5 Å^−1^ in the WT contour plots of materials can be assigned to the metal‐N coordination shell. Nevertheless, the WT signal of Ni atom in Ni→Mn DAC displays negative shift in wavevector *k* compared to the Mn‐N bond in Mn→Ni DAC, manifesting that the existence of Ni‐C coordination. These results verify that the axially coordinated Ni atoms are anchored by the C‐Ni‐N structure, while Mn atom achieves axial coordination by two N atoms. Furthermore, the electronic structure of DACs can be regulated by coordination environment engineering. The asymmetric C‐Ni‐N configuration can cause the electron rearrangement, which induced the different catalytic activities.

### 2.3 Electrocatalytic Performance for CO_2_RR

**Figure 4:**
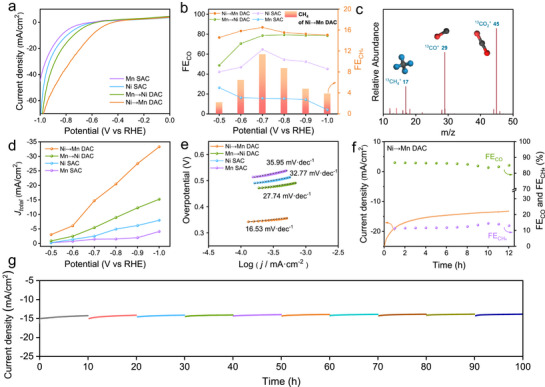
Eletrochemical performances for CO_2_ reduction. (a) LSV tests and (b) FE_CO_ or FE_CH4_ for all samples. (c) GC‐MS spectrum of reduction products for Ni→Mn DAC. (d) The *J*_total_ for all catalysts in 0.5 m KHCO_3_. (e) Tafel plots. (f) The stability measurement and (g) the long‐term cyclic stability of Ni→Mn DAC at −0.7 V (versus RHE).

The electrocatalytic CO_2_ reduction reaction (CO_2_RR) performance of all materials was investigated in CO_2_‐saturated 0.5 m KHCO_3_ electrolytes. The linear scanning voltammetry (LSV) curves reveal that all catalysts exhibit higher current responses under CO_2_ atmosphere compared to those under N_2_, demonstrating their catalytic activities for CO_2_RR (Figure S6, Supporting Information). Notably, Ni→Mn DAC presents much higher current response than that of Mn→Ni DAC and Ni/Mn SAC, indicating the superior activity of Ni→Mn DAC for CO_2_ reduction (**Figure** **4**a; Figure S7, Supporting Information). Furthermore, Ni→Mn DAC displays a maximum faraday efficiency of CO (FE_CO_) up to 86.6% at −0.7 V (vs RHE) in Figure 4b, surpassing that of Mn→Ni DAC (78.4% at −0.7 V), Ni SAC (64.7% at −0.7 V) and Mn SAC (26.7% at −0.5 V) (Equation S3.2, Supporting Information). Significantly, Ni→Mn DAC exhibits an extra CH_4_ product with 11.4% FE_CH4_ at −0.7 V. To clarify the source of CH_4_ produced by Ni→Mn DAC, we performed ^13^CO_2_ isotopic labeling experiments. The corresponding signals at m/z = 45 (^13^CO_2_), 29 (^13^CO) and 17 (^13^CH_4_) were detected when ^13^C‐labeled CO_2_ was used as the sole carbon source (Figure 4c). This result confirms that the CH_4_ originated from CO_2_ conversion. However, Mn→Ni DAC only increases the catalytic activity for single product CO in comparison with Ni SAC. As shown in Figure 4d and Figure S8a (Supporting Information), Ni→Mn DAC shows a CO partial current density (*J*_CO_) of 12.7 mA·cm^−2^ at −0.7 V with a *J*_CH4_ of 1.7 mA·cm^−2^ (Equation S3.3, Supporting Information), much higher than those of other control materials. For all tested catalysts, CO and H_2_ (as well as CH_4_ of Ni→Mn DAC) are the main products with the total FE close to 100% in the applied potentials and no liquid products were detected in NMR spectra (Figure S8c,d, Supporting Information).^[^ ^32^ ^]^

Ni→Mn DAC demonstrates smaller Tafel slope (101 mV dec^−1^) than Mn→Ni DAC (110 mV dec^−1^), Ni SAC (165 mV dec^−1^) and Mn SAC (196 mV dec^−1^) (Equation S3.1, Supporting Information), illustrating a faster kinetics for CO_2_RR (Figure 4e). Meanwhile, Ni→Mn DAC also displays the smallest charge transfer resistance in electrochemical impedance spectroscopy (EIS), further supporting its excellent catalytic activity (Figure S8b, Supporting Information). Furthermore, the double‐layer capacitance (*C*_dl_) for Ni→Mn DAC (26.5 mF·cm^−2^) is obviously higher those of Mn→Ni DAC (23 mF·cm^−2^), Ni SAC (14.9 mF·cm^−2^) and Mn SAC (9.6 mF·cm^−2^), indicating a larger electrochemical active surface area for Ni→Mn DAC (Figure S7, Supporting Information). Moreover, Ni→Mn DAC can be continuously operated at −0.7 V for 12 h with almost unchanged current density and FE_CO/CH4_ in Figure 4f. This exhibits its ultrahigh stability for CO_2_RR, further proved by the long‐term cyclic stability (Figure 4g). In addition, the Ni→Mn DAC retained excellent stability under both prolonged cycling tests and high‐voltage operation (Figure S8e,f, Supporting Information). The excellent CO_2_RR activities of Ni→Mn DAC compared to Mn→Ni DAC and Ni/Mn SAC with Ni or Mn single‐atoms only, manifesting that the axial coordinated Ni atoms effectively regulate Mn active sites of Ni→Mn DAC.

Furthermore, the morphology and chemical states of the catalysts after long‐term stability test were characterized to verify their robust durability. As shown in Figure S9a–c (Supporting Information), the DACs maintained fundamental cubic morphology after prolonged electrolysis with no observable clusters or particles resulting from metal detachment, which is consistent with the post‐reaction R‐space data (Figure S9e,f, Supporting Information). The STEM images still show individually dispersed bright atomic spots (Figure S9c, Supporting Information), confirming that no aggregation of single‐atoms occurred. Additionally, the distinct signals of Ni and Mn were detected by XPS (Figure S9g,h, Supporting Information), demonstrating that the single‐atoms remain stably anchored on the support. This results confirm the stable atomic anchoring of metal species on the support. The nearly identical I_G_/I_D_ ratios in the Raman spectra (Figure S9d, Supporting Information) before and after reaction demonstrate preserved graphitization degree and absence of newly formed species, further verifying the stable anchoring of single‐atoms. Notably, XPS spectra (Figure S10a, Supporting Information) still distinctly resolved the axially coordinated metal elements in the DACs, demonstrating the excellent structural stability. Furthermore, compared with other reported catalysts in Table S4, the present catalyst demonstrates remarkable performance by maintaining a high Faradaic efficiency for CO while simultaneously generating CH_4_ as an additional valuable product. Collectively, the DACs exhibit superior stability.

### 2.4 Confirmation of Electron and Spin States for Metal Active Sites

**Figure 5:**
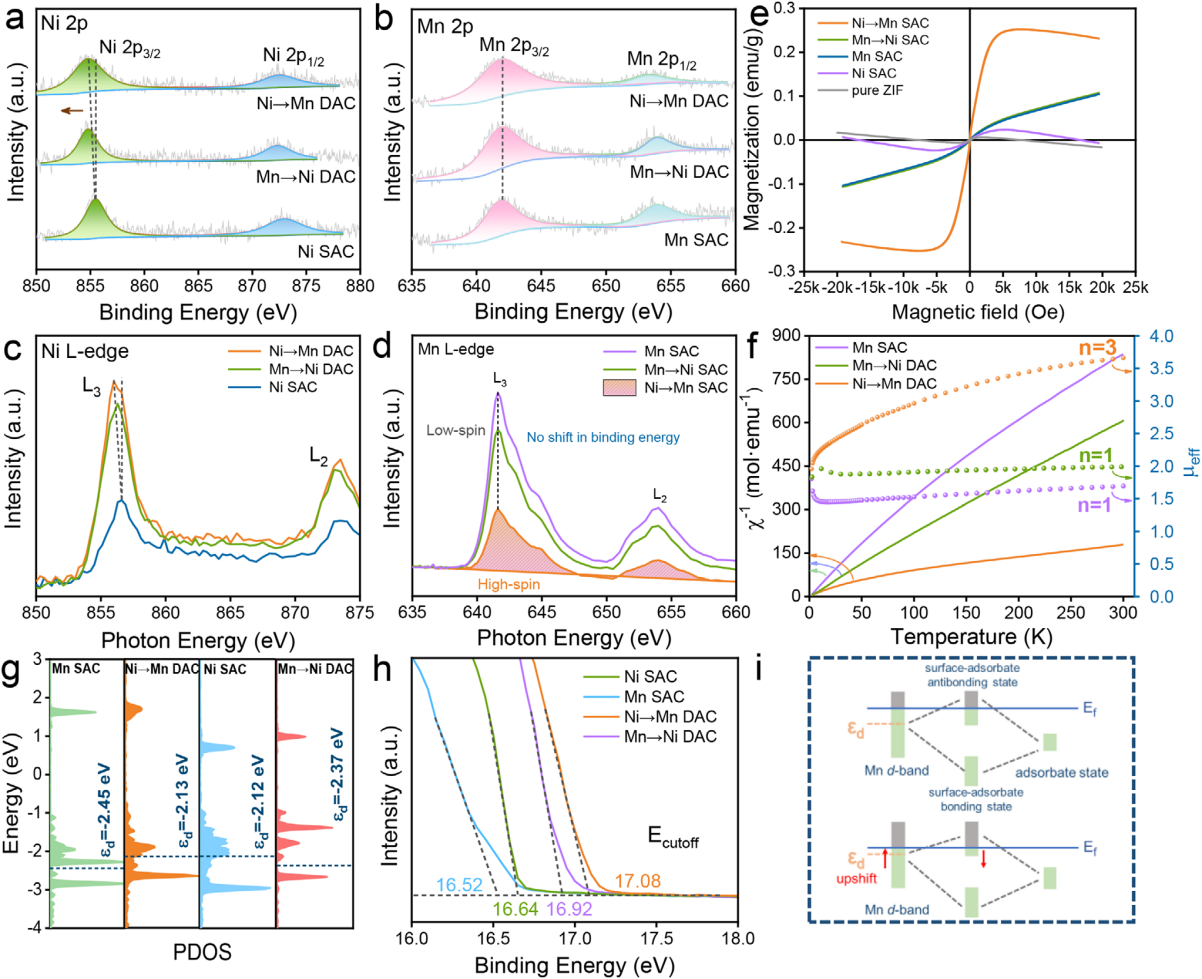
Electron spin state characterization. High‐resolution XPS spectra of a) Ni 2*p* and b) Mn 2*p*. c) Ni *L*‐edge and d) Mn *L*‐edge XANES spectra. e) VSM curves. f) 1/χ plots and the µ_eff_. g) The *d*‐band center spectra. h) The UPS spectra. i) Schematic of *d*‐band center upshift.

The above results show that Ni→Mn DAC enables robust CO_2_ reduction with accelerating the generation of valuable CH_4_, which could be attributed to the variation of spin states. As illustrated in **Figure** **5**a, the core‐level scan XPS spectra of Ni 2*p* comprise two main peaks belonging to Ni 2*p*_3/2_ and Ni 2*p*_1/2_, which resulted from the spin‐orbit splitting of the *p* orbital.^[^ ^33^ ^]^ The valence state of Ni SAC is confirmed to be +2 by comparison with the standard samples (Figure S10b, Supporting Information), which is consistent with the Ni *K*‐edge XANES result. These two peaks shift to lower binding energies for dual‐metal single‐atoms compared to Ni SAC, indicating the reduction of valence state.^[^ ^34^, ^35^ ^]^ In contrast, the two main peaks located at 642.1 (Mn 2*p*_3/2_) and 653.9 eV (Mn 2*p*_1/2_) exhibit negligible shifts for Mn‐related samples in Mn 2*p* XPS spectra (Figure 5b; Figure S10b, Supporting Information), which implies that the valence state of Mn^4+^ remains unchanged. The XPS results of metal valence states are consistent with the XANES data (Figure 3a,b). To further elucidate the electron distribution in the catalysts, we performed differential charge density analysis for Ni→Mn DAC and Mn→Ni DAC. As shown in Figure S13 (Supporting Information), the Mn active sites in Ni→Mn DAC show negligible electron transfer, whereas the Ni sites in Mn→Ni DAC clearly gain electrons, leading to a reduced valence state of Ni. This result is fully consistent with the XPS data. Additionally, Ni/Mn dopants exhibit apparent signals before and after the reaction (Figure S10a, Supporting Information), suggesting that the introduced metals were stably anchored inside the materials.

Based on the above results, the metal soft XANES was performed to explore the subtle change in electronic state. As shown in Figure 5c, the Ni *L*‐edge spectra are dominated by the Ni 2*p*→3*d* transition and split into two peaks located at 856.3 and 873.3 eV, corresponding to the *L_3_* and *L_2_* edges, respectively.^[^ ^36^ ^]^ The characteristic peaks of Ni‐related materials shift to lower binding energies, in accordance with the XPS and XANES results, suggesting that Ni atom within dual‐metal single‐atoms catalysts accepts electron. However, the Mn *L_3_*‐edge spectra show main peaks (641.6 and 653.9 eV) without an obvious shoulder, indicating that only Mn^4+^ species exist in Mn‐related catalysts. Notably, the shape of the spectrum strongly relies on the structure, which derived from the hybridization of Mn 3*d* orbitals with ligand N 2*p* orbitals. Therefore, the shape and intensity of *L_3_*‐edge peak reflect the information of metal spin states, from which the absorption peak intensity of *L_3_*‐edge directly manifests the unoccupied states of active metal 3*d* orbitals.^[^ ^37^ ^]^ The decreasing intensity of Ni→Mn DAC implies more Mn 3*d* orbitals should be filled, transforming into a high spin state of Mn^4+^. Compared with Mn SAC, the Ni axial coordination modulates the transition of Mn spin state from low‐spin to high‐spin.

To further investigate the magnetic properties and spin configurations of Mn‐related samples, vibrating sample magnetometer (VSM) was employed. As shown in Figure 5e, Mn‐related materials display paramagnetic behavior, especially Ni→Mn DAC shows superparamagnetic property, confirming the existence of spin electrons. In addition, Ni SAC exhibits diamagnetic character, which means that electrons are arranged in pairs in Ni 3*d* orbital. The calcined ZIF‐8 support exhibits predominantly diamagnetic behavior, thereby ruling out its contribution to the magnetic properties of the final catalysts. The result was further verified by the electron paramagnetic resonance (EPR) data in Figure S10c (Supporting Information). In order to reveal the electron spin configuration of active metal sites, the superconducting quantum interference device (SQUID) was performed. From the 1/χ plots (Figure 5f), the axial coordination of Ni atom increases the paramagnetic state of Mn species, exhibiting that more free electrons with Pauli paramagnetism were arranged in Mn 3*d* orbital. According to the equation $2.828\sqrt{\chi T\;}=\mu_{\mathrm{eff}}=\sqrt{n(n+2)}$,^[^ ^38^, ^39^ ^]^ the number of unpaired Mn 3*d* electrons (n) related to the effective magnetic moment (µ_eff_) can be calculated. Apparently, Ni→Mn DAC exhibits more unpaired spin electrons (≈3) in comparison with Mn SAC (≈1), verifying the transformation of low‐spin to high‐spin. Moreover, Ni SAC shows no unpaired spin electrons in Figure S10d, which is consistent with VSM and EPR results.

To further investigate the influence of changes in the electronic spin configuration of catalyst on the adsorption of intermediates, the *d*‐band theory was employed to examine the binding of adsorbates between the transition metal and intermediate species.^[^ ^40^ ^]^ In Figure 5g, compared with Mn SAC (−2.45 eV), the *d*‐band center (ε_d_) of Mn 3*d* orbits for Ni→Mn DAC (−2.13 eV) shifts to a higher energy level. The antibonding states of Ni→Mn DAC configuration and adsorbed species are less occupied (Figure 5i), thereby moderately increasing the binding interaction of intermediates.^[^ ^41^ ^]^ Hence, the Mn active sites of Ni→Mn DAC strongly adsorb ^*^CO intermediates to further generate CH_4_. In contrast, the Ni 3*d* orbital ε_d_ of Mn→Ni DAC (−2.37 eV) is lower than that of Ni SAC (−2.12 eV), leading to the weak interaction between Ni active sites and intermediates. Thus, Mn→Ni DAC is tends to desorb ^*^CO intermediates, resulting in a higher FE_CO_ than that of Ni SAC, which is in good agreement with the CO_2_RR performance. This is further confirmed by ultraviolet photoelectron spectroscopy (UPS) in Figure 5h, which was performed to understand the band structure information. The cutoff energy (E_cutoff_) of Ni→Mn DAC and Mn→Ni DAC is 17.08 and 16.92 eV, respectively. According to the equation of e_ϕ_ = 21.22 eV −E_cutoff_,^[^ ^42^, ^43^ ^]^ the work functions (e_ɸ_) of Ni→Mn DAC and Mn→Ni DAC are calculated as 4.14 and 4.3 eV, respectively. This rendering that Ni→Mn DAC tends to donate more electrons to intermediates, which can facilitates the reduction process.

### 2.5 Theoretical calculations of Electron and Spin States of Metal Active Sites

**Figure 6:**
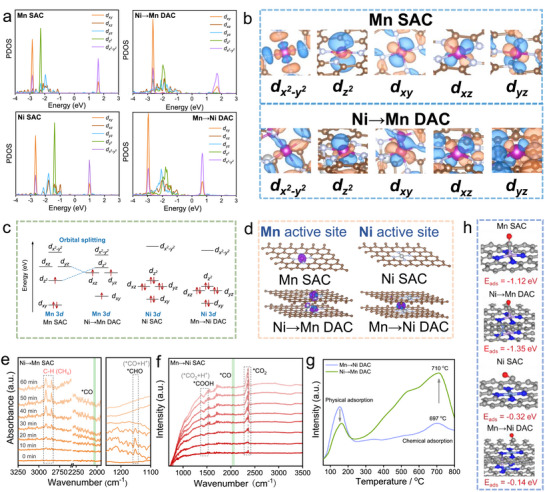
The theoretical CO_2_RR mechanism. a) The PDOS spectra for all catalysts. b) The wave functions of Mn 3*d* orbitals with corresponding energy level labels. c) 3*d* orbital diagrams. d) The corresponding spin density diagram. The in situ ATR‐FTIR for e) Ni→Mn DAC and f) Mn→Ni DAC. g) CO‐TPD patterns collected for Ni→Mn DAC and Mn→Ni DAC. h) The calculated ^*^CO adsorption energy for all catalysts.

To provide an in‐depth investigation for the intrinsic properties variation, density functional theory (DFT) theoretical calculations and experimental examinations were further conducted. According to the projected density of states (PDOS) spectra and the orbitals originated from the wave functions for all catalysts (**Figure** **6**a,b; Figure S11, Supporting Information), the 3*d* orbital models of metal active sites were calculated as shown in Figure 6c. With the axial coordination of Ni atoms, Ni→Mn DAC exhibits obvious orbital splitting compared to Mn SAC, resulting in the redistribution of Mn 3*d* orbital electrons. This mainly originates from the decreasing degenerate orbital energy level of *d_xz_* and *d_yz_*, leading to the increasing electronic spin state of Mn 3*d* ^3^. Therefore, Ni atoms modulate the spin state of Mn 3*d* ^3^ from low‐spin state (*d_xy_* ↑↓ *d_z_^2^* ↑) transform to high‐spin state (*d_xy_* ↑ *d_xz_* ↑ *d_yz_* ↑), in accordance with the magnetic experimental data. On the contrary, the arrangement of 3*d* orbital energy levels and the electronic spin states for Ni active sites exhibit ignored change, which explains the essential factor for the better catalytic performance of Ni→Mn DAC. Furthermore, the increasing spin state can be visualized by the corresponding spin density diagram (Figure 6d), where Mn active site demonstrates higher spin state for Ni→Mn DAC with a wider spin‐related channel. As a result, Ni→Mn DAC promotes the electron transport and donation during CO_2_RR, further accelerating the reaction kinetics.

To identify the key intermediates during the CO_2_ reduction, in situ attenuated total reflection Fourier transform infrared (ATR‐FTIR) spectroscopy was adopted. As depicted in Figure 6g, the small peak at 2033 cm^−1^ can be ascribed to stretching vibration of linear CO from CO_2_, implying the consumption of adsorbed CO_2_. The positive peak located at 1151 cm^−1^ is attributed to ^*^CHO,^[^ ^44^, ^45^ ^]^ which is defined as the crucial intermediate for generating CH_4_. Significantly, the obvious signals of the asymmetric (2916 cm^−1^) and symmetric (2848 cm^−1^) C─H stretching vibration gradually strengthen, demonstrating the gradual evolution of CH_4_ on the Ni→Mn DAC surface.^[^ ^46^, ^47^, ^48^ ^]^ However, only the process of Mn→Ni DAC producing CO is observed. As depicted in Figure 6h, the peak at 1431 cm^−1^ related to ^*^COOH and the peak at 2047 cm^−1^ referred to CO emerge and strengthen gradually,^[^ ^14^, ^49^, ^50^ ^]^ verifying the production of the typical ^*^COOH intermediate for reducing CO_2_ to CO. The in situ ATR‐FTIR experimental results are directly prove the generation of related products, which is in agreement with the theoretical calculation of PDOS and *d*‐band center. To further confirm the strong adsorption of ^*^CO on Ni→Mn DAC, carbon monoxide temperature‐programmed desorption (CO‐TPD) experiments were conducted. As shown in Figure 6g, Mn→Ni DAC exhibits a strong desorption peak of physically adsorbed CO at ≈150 °C, while a very weak chemisorbed CO desorption peak appears at 697 °C. This indicates that Mn→Ni DAC has limited capability for ^*^CO chemisorption, consistent with the downshift of Ni *d*‐band center (Figure 5g). In contrast, Ni→Mn DAC shows a much weaker physisorption peak, but a notably stronger chemisorbed CO desorption peak at 710 °C. Compared to Mn→Ni DAC, Ni→Mn DAC exhibits a CO chemisorption peak at a higher temperature with a greater intensity, demonstrating its enhanced ability to strongly adsorb ^*^CO. This can further facilitate hydrogenation toward CH_4_ formation.

Furthermore, adsorption energy calculations revealed that Ni→Mn DAC (−1.35 eV) has a higher affinity for ^*^CO adsorption compared to Mn→Ni DAC (−0.14 eV), Ni SAC (−0.32 eV), and Mn SAC (−1.12 eV). Conversely, Mn→Ni DAC favors ^*^CO desorption, which aligns with the experimental observation that Mn→Ni DAC cannot further hydrogenate adsorbed ^*^CO to CH_4_ (Figure S12a, Supporting Information). In addition, the Gibbs free energy profiles for the key intermediates along the CH_4_ formation pathway were calculated (Figure S12b, Supporting Information). Notably, the Ni→Mn DAC exhibits the lowest activation barrier for the critical ^*^CO→^*^CHO step among all Mn‐related catalysts. This lower energy barrier, combined with its enhanced adsorption of key carbon‐containing intermediates, promotes the deep hydrogenation of ^*^CO, leading to the selective formation of CH_4_. Both experimental and computational results consistently demonstrate that the strong ^*^CO adsorption on Ni→Mn DAC is crucial for enabling its subsequent conversion to CH_4_. Consequently, the axial coordinated Ni atom (Ni→Mn DAC) manipulates Mn active site from low‐spin state to high‐spin state through orbital splitting, which is conducive to donating electrons and thus accelerating the reaction kinetics. Meanwhile, Ni→Mn DAC enhances the adsorption of the key ^*^CO intermediate, thereby further rapidly generate CH_4_ based on the accelerated reaction dynamics.

## 3 Conclusion

In summary, we synthesized Ni→Mn DAC and Mn→Ni DAC materials by double‐solvent method, in which the introduced metal shows axial coordination around the active site. This results in diverse coordination environment of two samples, suggesting the distinct electronic structures. Significantly, the axial coordinated Ni atoms in Ni→Mn DAC drive the spin‐state transition of the Mn active sites from the low‐spin state to the high‐spin state by inducing Mn 3*d* orbital splitting. Moreover, the upshifted Mn *d*‐band center indicates a strong adsorption between the Mn active site and the ^*^CO key intermediate. Based on the rapid dynamics, the eight‐electron process of producing CH_4_ is fast accomplished. In contrast, Mn→Ni DAC displays the downshifted Ni *d*‐band center and negligible change of electronic spin state, promoting the desorption of key intermediates and thus increasing CO_2_RR property of evolving single product CO.

## Conflict of Interest

The authors declare no conflict of interest.

## Supporting information



Supporting Information

## Data Availability

The data that support the findings of this study are available from the corresponding author upon reasonable request.
