# Supplementary material for: Manipulating Spin States by Metal Axial Coordination of Active Sites for Generating Valuable CH4 in CO2 Reduction
Source: Adv Sci (Weinh). 2025 Nov 6;13(4):e17166. doi: 10.1002/advs.202517166 (PMC12822474; doi:10.1002/advs.202517166)
Supplement: Supplementary file 1 — Supporting Information [file ADVS-13-e17166-s001.docx]

Supporting information

**Manipulating spin states by metal axial coordination of active sites for generating valuable CH_4_ in CO_2_ reduction**

Min Zhang^1^, Qi Zhao^1^, Yixuan Gao^2^, Lirong Zheng^3^, Jin Ouyang^4^ and Na Na*^1^

^1^Key Laboratory of Radiopharmaceuticals, Ministry of Education, College of Chemistry, Beijing Normal University, Beijing 100875, China

^2^State Environmental Protection Key Laboratory of All Material Fluxes in River Ecosystems, College of Environmental Sciences and Engineering,

Peking University, Beijing, 100871, China

^3^Beijing Synchrotron Radiation Facility Institute of High Energy Physics Chinese Academy of Sciences, Beijing 100049, China

^4^Department of Chemistry, College of Arts and Sciences, Beijing Normal University at Zhuhai, Zhuhai, 519087, China

* Corresponding Author: [nana@bnu.edu.cn](mailto:nana@bnu.edu.cn)

**Table of content**

[Section 1. Chemicals and Instrumentation 1](#_Toc171620315)

[1.1 Chemicals 1](#_Toc171620316)

[1.2 Instrumentation 1](#_Toc171620317)

[Section 2. Experimental methods 2](#_Toc171620318)

[2.1 Synthesis of Ni ZIF and Mn ZIF 2](#_Toc171620319)

[2.2 Synthesis of Ni→Mn ZIF and Mn→Ni ZIF with double-solvent method 2](#_Toc171620321)

[2.3 Synthesis of Ni SAC, Mn SAC, Ni→Mn DAC and Mn→Ni DAC 2](#_Toc171620323)

[Section 3. Detailed calculation procedures 2](#_Toc171620325)

[3.1 Tafel slope 2](#_Toc171620326)

[3.2 Calculation of Faradaic efficiency 3](#_Toc171620329)

[3.3 Calculation of the CO and CH_4_ partial current density 3](#_Toc171620334)

[Section 4. XAFS measurement and data analysis 3](#_Toc171620337)

[Section 5. Electrochemical measurements 4](#_Toc171620338)

[Section 6. Computational methods and models 5](#_Toc171620339)

[Section 7. Supplementary Figures and Tables 6](#_Toc171620340)

Section 1. Chemicals and Instrumentation

1.1 Chemicals

All chemicals were purchased from commercial sources and used without further purification. ZnSO_4_·7H_2_O, MnSO_4_·H_2_O and 2-Methylimidate were purchased from Shanghai Macklin Reagent Co. Ltd. NiCl_2_, Hexadecyltrimethyl ammonium bromide (CTAB), Anhydrous ethanol and dimethyl sulfoxide (DMSO) were provided by Shanghai Aladdin Reagent Co. Ltd. Acetylene black, n-hexane and proton exchange membrane (Nafion211) were purchased from Beijing Innochem Reagent Co. Ltd. Soft hydrophilic carbon cloth (HCP020N) and Nafion (D520, 5 %) were provided by Shanghai Hesen Electric Co., LTD. High-purity water (18.2 MΩ·cm) supplied by Mill-Q Purification System.

1.2 Instrumentation

The field emission scanning electron microscope (FESEM) (Model SU-8010, Japan) was adopted for imaging. The high-resolution transmission electron microscopy (HRTEM) of FEI Talos 200s TEM was applied for characterizations at an operating voltage of 200 kV. High-angle annular dark field scanning transmission electron microscopy (HAADF-STEM) measurements were taken on a transmission electron microscopy with a probe corrector (FEI Theims Z, Titan Cubed Themis G2300, JEM-ARM200F). X-ray diffraction measurement was characterized by Maxima XRD-7000 (Shimadzu, Japan). XPS spectra were recorded by using an X-ray spectrometer (Thermo ESCALAB 250 XI, PHI-5000 versaprobe III, Thermo Kalpha) with Al Kα excitation source. Raman spectra were recorded by Renishaw RM-1000 Raman spectrometer at room temperature, with 532 nm laser as excitation light source. The production of CO, CH_4_ and H_2_ were detected by gas chromatography (GC, 2014C, Ar carrier, Shimadzu). XAFS spectra at the Ni (8333 eV) and Mn (6539 eV) K-edge were measured at the 1W1B beamline of the Beijing Synchrotron Radiation Facility (BSRF) and BL14W1 beamline of the Shanghai Synchrotron Radiation Facility (SSRF), respectively. The XANES spectra at the Ni/Mn L-edge and C/N K-edge were measured at National Synchrotron Radiation Laboratory (NSRL) in Hefei. The EPR spectra were obtained using Bruker EMXplus spectrometer.

Section 2. Experimental methods

2.1 Synthesis of Ni ZIF and Mn ZIF

2.7 g 2-methylimidazole and 3 mg CTAB were dissolved in 50 mL water under continuously stirring conditions. Then 175 mg ZnSO_4_ and 3 mg NiCl_2_ were added into in turn. After stirring for another 6 h, the resultant mixture was centrifuged (10000 r/min) and washed with enough ethanol and H_2_O respectively to obtain the Ni-ZIF. Mn ZIF was prepared with the same method of Ni-ZIF but using MnSO_4_ instead of NiCl_2_.

2.2 Synthesis of Ni→Mn ZIF and Mn→Ni ZIF with double-solvent method

The powder of Mn ZIF (100 mg) was dispersed in n-hexane (12 mL) under ultrasound for 1 h at room temperature. After forming a homogeneous suspension, NiCl_2_ aqueous solution (30 mg·mL^-1^, 100 μL) was added dropwise to the above solution under stirring within 5 min. Next, the mixed solution was stirred for another 2 h at room temperature to obtain Ni→Mn ZIF. Then, the material was centrifuged, washed with enough ethanol several times to remove n-hexane, and dried under vacuum at 60 ^o^C overnight. Mn→Ni ZIF was prepared with the same method of Ni→Mn ZIF but using Ni ZIF and MnSO_4_ instead of Mn ZIF and NiCl_2_, respectively.

2.3 Synthesis of Ni SAC, Mn SAC, Ni→Mn DAC and Mn→Ni DAC

The Ni ZIF, Mn ZIF, Ni→Mn ZIF and Mn→Ni ZIF pyrolyzed in an aluminum oxide ceramic boat in a tube furnace. The temperature was increased to 900 ^o^C for 2 h with a heating rate of 5 ^o^C/min to synthesize Ni SAC, Mn SAC, Ni→Mn DAC and Mn→Ni DAC under flowing N_2_ and then naturally cooled down to the room temperature. All samples were washed with 0.5 M HCl overnight after pyrolysis to remove any possible nanoparticles or clusters.

Section 3. Detailed calculation procedures

3.1 Tafel slope

$$\boldsymbol{\eta}\boldsymbol{=a+blogI}$$

where η represents the overpotential, a and b are the Tafel constant, I represents the current density (mA·cm^-2^).

3.2 Calculation of Faradaic efficiency

The faradaic efficiency of CO, CH_4_ and H_2_ were calculated as below:

$$\boldsymbol{FE=}\frac{\boldsymbol{Q}_{\boldsymbol{CH}_{\boldsymbol{4}}\boldsymbol{/CO/}\boldsymbol{H}_{\boldsymbol{2}}}}{\boldsymbol{Q}_{\boldsymbol{total}}}\boldsymbol{\times100\%=}\frac{\boldsymbol{z}\boldsymbol{\times F\times}\boldsymbol{n}_{\boldsymbol{CH}_{\boldsymbol{4}}\boldsymbol{/CO/}\boldsymbol{H}_{\boldsymbol{2}}}}{\boldsymbol{I\times t}}\boldsymbol{\times100\%}$$

$$\boldsymbol{n}_{\boldsymbol{CH}_{\boldsymbol{4}}\boldsymbol{/CO/}\boldsymbol{H}_{\boldsymbol{2}}}\boldsymbol{=}\frac{\boldsymbol{P\times V}_{\boldsymbol{CH}_{\boldsymbol{4}}\boldsymbol{/CO/}\boldsymbol{H}_{\boldsymbol{2}}}}{\boldsymbol{R\times T}}$$

where z represents the number of electrons transferred to CO, H_2_ (z = 2) and CH_4_ (z = 8); F is the Faradaic constant (96485 C·mol^-1^); n represents the mole numbers of CO, CH_4_ and H_2_, I is the average current over a test period (t), P is the atmospheric pressure (101.3 kPa), R is the ideal gas constant (8.314 J·mol^-1^·K^-1^), T is the room temperature (298.15 K), V is the volume of CO_2_.

3.3 Calculation of the CO and CH_4_ partial current density

$$\boldsymbol{J}_{\boldsymbol{CO/}\boldsymbol{CH}_{\boldsymbol{4}}}\boldsymbol{=}\boldsymbol{J}_{\boldsymbol{t}\boldsymbol{otal}}\boldsymbol{\times}\boldsymbol{FE}_{\boldsymbol{CO/}\boldsymbol{CH}_{\boldsymbol{4}}}$$

where *J*_CO/CH4_ is the CO or CH_4_ partial current density (mA·cm^-2^), *J*_total_ is the total current density.

Section 4. XAFS measurement and data analysis

The EXAFS spectra were obtained by subtracting the post-edge background from the overall absorption and then normalizing with respect to the edge-jump step. And then, the χ(k) data was Fourier transformed to real R-space to separate the EXAFS contributions from different coordination shells. To confirm the quantitative structural parameters around central atoms, least-squares curve parameter fitting was performed using the Artemis module. The following EXAFS equation was used for the calculation of the theoretical scattering amplitudes, phase shifts and the photoelectron mean free paths.

$$X\left(k\right)=\sum_{j} \frac{N_{j}S_{O}^{2}F_{j}\left(k\right)}{kR_{j}^{2}}\exp\left[-2k^{2}\sigma_{j}^{2}\right]\exp\left[\frac{-2R_{j}}{\lambda(k)}\right]\sin\left[2kR_{j}+\Phi_{j}(k)\right]$$

R_j_ is the distance between the X-ray absorbing central atom and the atoms in the j^th^ atomic shell, N_j_ is the number of neighbors in the j^th^ atomic shell, S_0_^2^ is the amplitude reduction factor, F_j_(k) is the effective curved-wave backscattering amplitude, λ is the mean free path in Å, ϕ_j_(k) is the phase shift (including the phase shift for each shell and the total central atom phase shift), σ_j_ is the Debye-Waller parameter of the j^th^ atomic shell (variation of distances around the average R_j_). All fits were performed in the *R* space with *k*^2^-weight for Ni and Mn K-edge.

Section 5. Electrochemical measurements

The CO_2_RR measurements were performed by electrochemical workstation (CH Instruments 660D). The electrolyte was CO_2_-saturated 0.5 M KHCO_3_ (pH 7.2). The CO_2_RR performance of the prepared catalysts are tested via a typical H-type cell, in which the catalysts (Ni SAC, Mn SAC, Ni→Mn DAC and Mn→Ni DAC) as the working electrode, a saturated Ag/AgCl electrode as the reference electrode and a Pt foil (1×1 cm^2^) as the counter electrode. The working electrode was prepared by dispersing 5 mg of catalyst and 30 μL of 5% Nafion solution in 700 μL ethanol and 300 μL deionized water with sonicating for 30 min to form a uniform ink. Then the catalyst ink was dropped onto a 1×1 cm^2^ carbon cloth.

According to the Nernst equation, the measured potentials vs. Ag/AgCl were converted to the reversible hydrogen electrode (RHE) scale:

$$E_{RHE}=E_{Ag/AgCl}+0.059pH+E_{Ag/AgCl}^{\theta}$$

Where $E_{Ag/AgCl}^{\theta}$ = 0.22 V at 25 ^o^C, $E_{RHE}$is the converted potential vs RHE, $E_{Ag/AgCl}$ is the experimentally measured potential against the Ag/AgCl reference.

The products were examined by GC with a thermal conductivity detector (TCD) for H_2_ and a flame ionization detector (FID) for CO and CH_4_. Liquid products were characterized by proton nuclear magnetic resonance (^1^H NMR) adopting DMSO as an internal standard.

Section 6. Computational methods and models

All geometry optimizations and energy calculations were executed by the Vienna ab initio Simulation Package (VASP 6.3) with the generalized gradient approximation (GGA) using the Perdew-Burke-Ernzerhof (PBE) formulation. The interaction between core electrons and valence electron using a plane wave basis set with the energy cutoff of 500 eV was described by the projected augmented wave (PAW) method. Due to the effect of van der Waals (vdW) interactions in graphite interlayer, the DFT-D3 method by Grimme was employed. Atomic structures were fully released and converging tolerance for forces on all atoms and the energy convergence criterion was set to 0.02 eV/Å and 10^-5^ eV. The Brillouin zone was sampled using a 3×3×1 Monkhorst-Pack k-meshes for geometry relaxation.

The CO_2_RR performance was explored under the theoretical framework developed by Nørskov *et al*. The associative mechanism and an eight-electron pathway were considered, according to which the CO_2_RR elementary reactions are described as follows:

$$\left( 1 \right) *CO_{2} + H^{+} + e^{-} = *COOH$$

$$\left( 2 \right)*COOH+ H^{+} + e^{-} = *CO+ H_{2}O$$

$$\left( 3 \right) *CO+ H^{+} + e^{-} = *CHO$$

$$\left( 4 \right) *CHO+ H^{+} + e^{-} = *CHOH$$

$$\left( 5 \right) *CHOH {+ H}^{+} + e^{-}= *CH_{2}OH$$

$$\left( 6 \right) *CH_{2}OH+H^{+} + e^{-} = *CH_{2}+ H_{2}O$$

$$\left( 7 \right) *CH_{2}+ H^{+} + e^{-} = *CH_{3}$$

$$\left( 8 \right) *CH_{3} + H^{+} + e^{-} = *CH_{4}$$

Where * represents an active site. *COOH, *CO, *CHO, *CHOH, *CH_2_OH, *CH_2_ and *CH_3_ are the active sites with COOH, CO, CHO, CHOH, CH_2_OH, CH_2_ and CH_3_ intermediate adsorption, respectively.

Section 7. Supplementary Figures and Tables


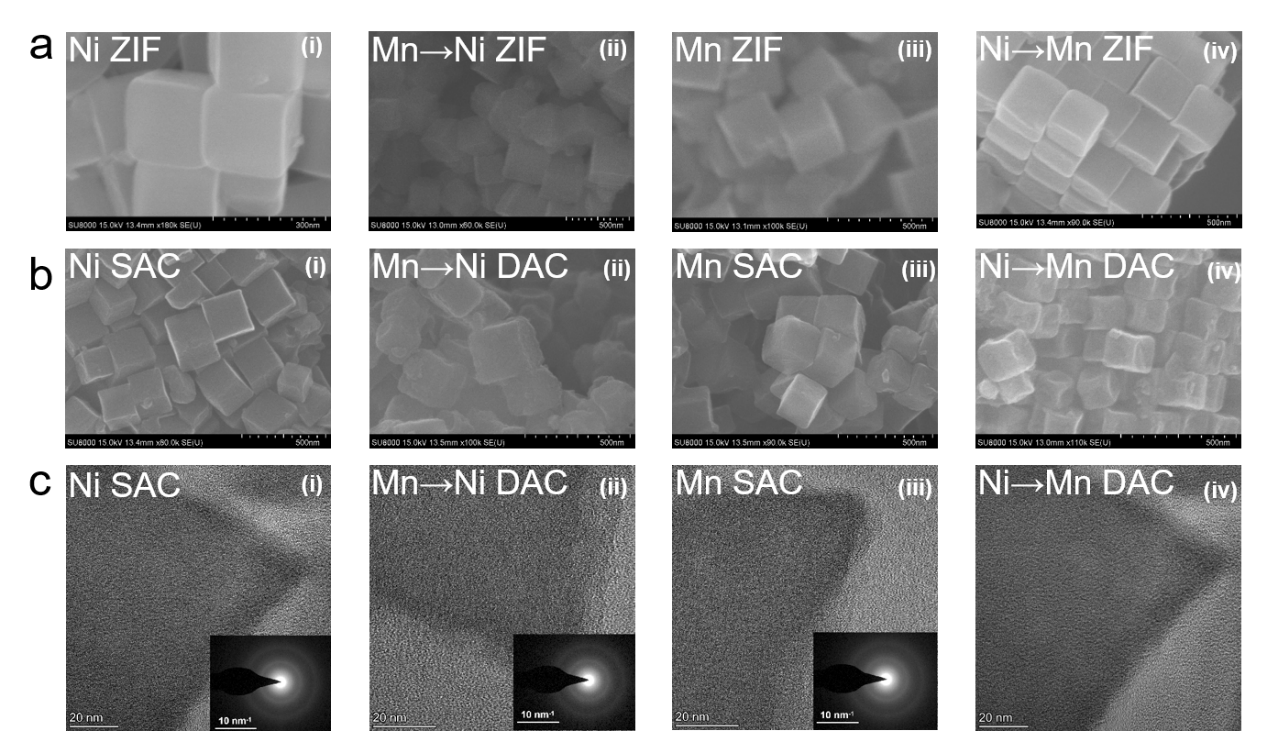


**Figure S1.** SEM images of (a) Ni/Mn ZIF, Mn→Ni ZIF and Ni→Mn ZIF, (b) Ni/Mn SAC, Mn→Ni DAC and Ni→Mn DAC. (c) The corresponding HRTEM images (inset: the corresponding SAED images).


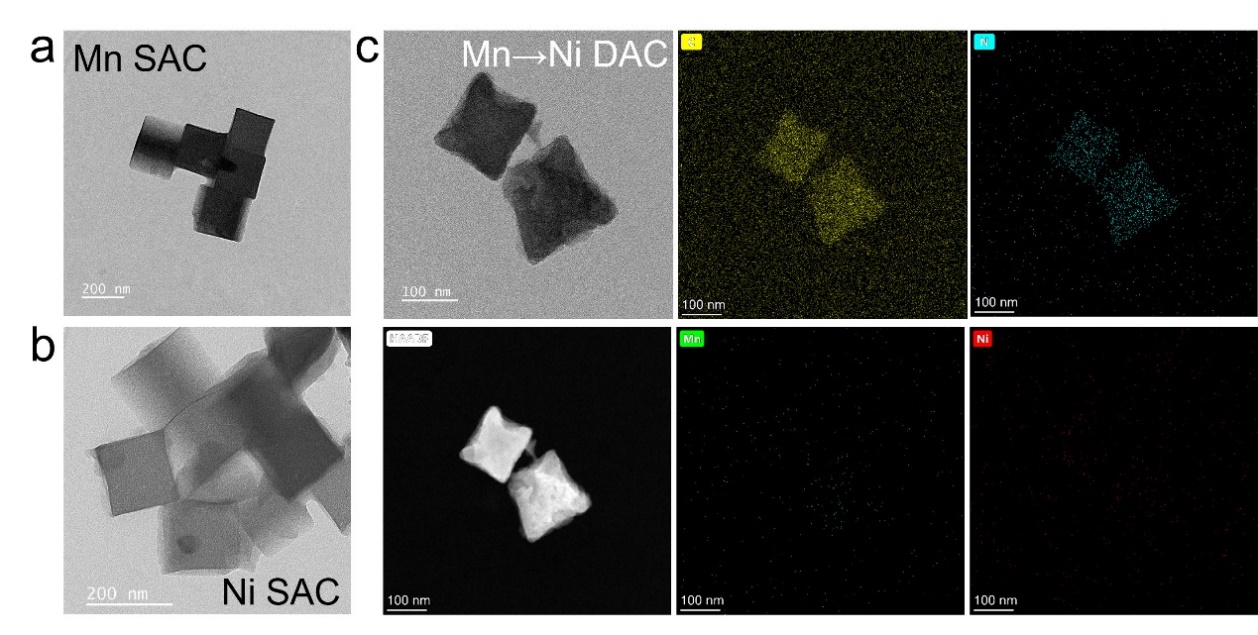


**Figure S2.** TEM images of (a) Mn SAC and (b) Ni SAC. (c) The corresponding EDS maps of Mn→Ni DAC.

**Figure S3.** The XRD patterns of (a) pure ZIF, Ni/Mn ZIF, Mn→Ni ZIF and Ni→Mn ZIF, (b) Ni/Mn SAC, Mn→Ni DAC and Ni→Mn DAC. (c) The Raman spectra for all catalysts. The N_2_ adsorption-desorption isotherm and the corresponding pore size distribution for (d) Mn ZIF, Ni→Mn ZIF, (e) Mn SAC and Ni→Mn DAC.


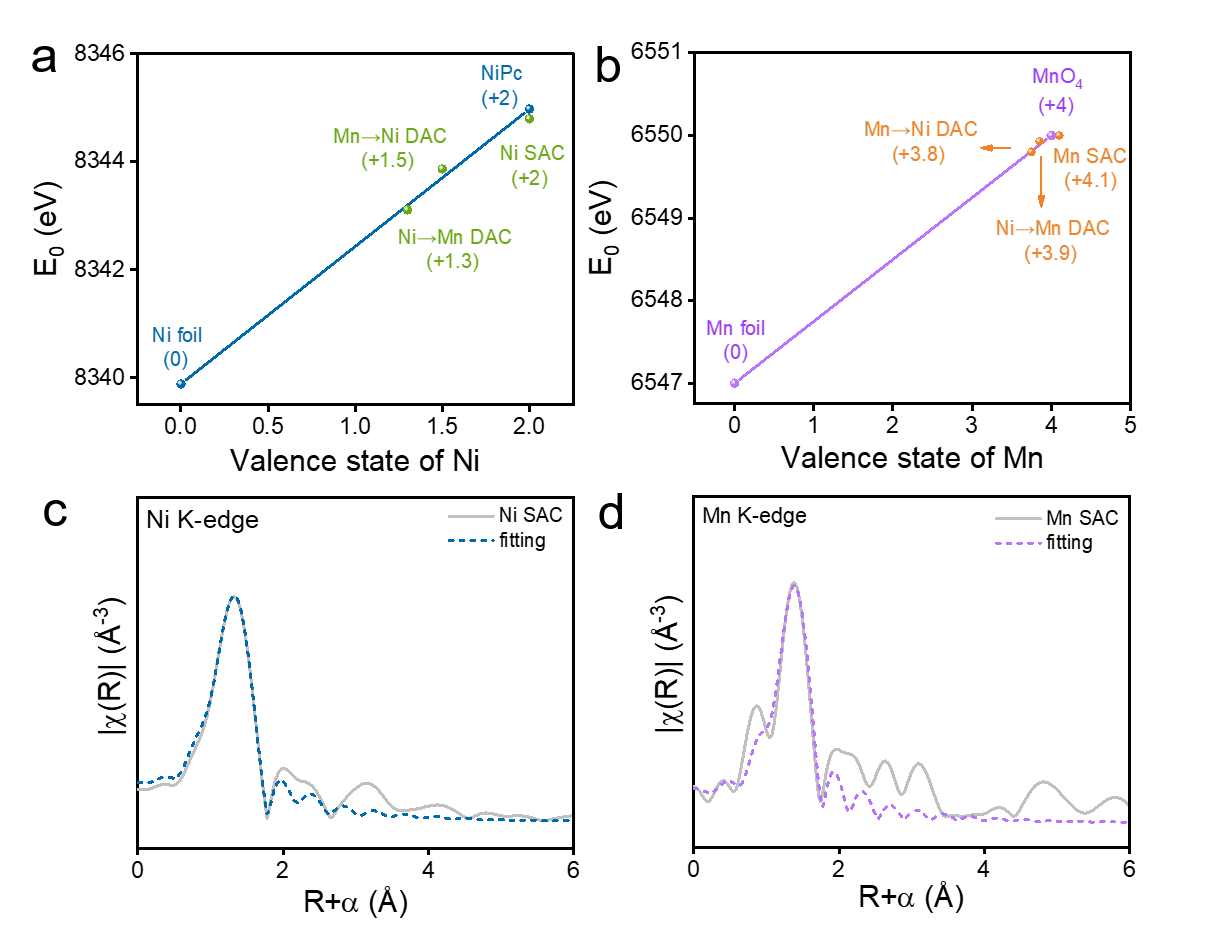


**Figure S4.** Calculated (a) Ni and (b) Mn valence state on operando Ni and Mn *K*-edge XAS by absorption edge. The corresponding EXAFS fitting results for (c) Ni SAC and (d) Mn SAC.


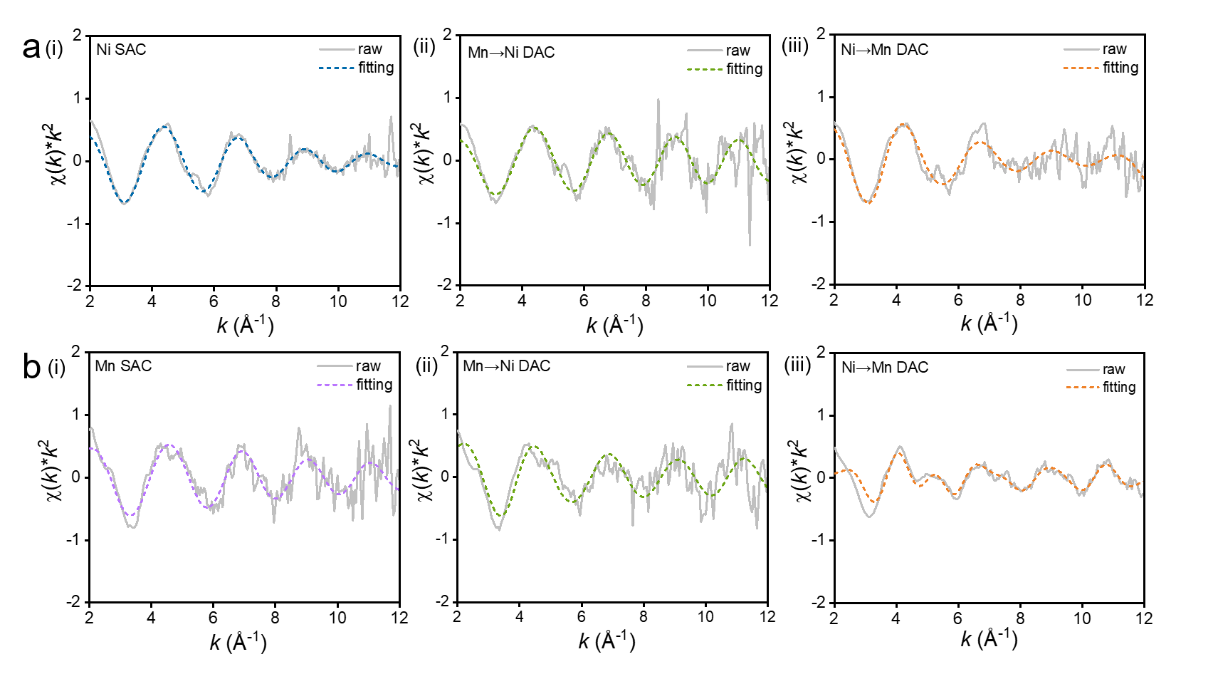


**Figure S5.** Model-based fitting results of operando EXAFS *k*-space of (a) Ni-related and (b) Mn-related catalysts.


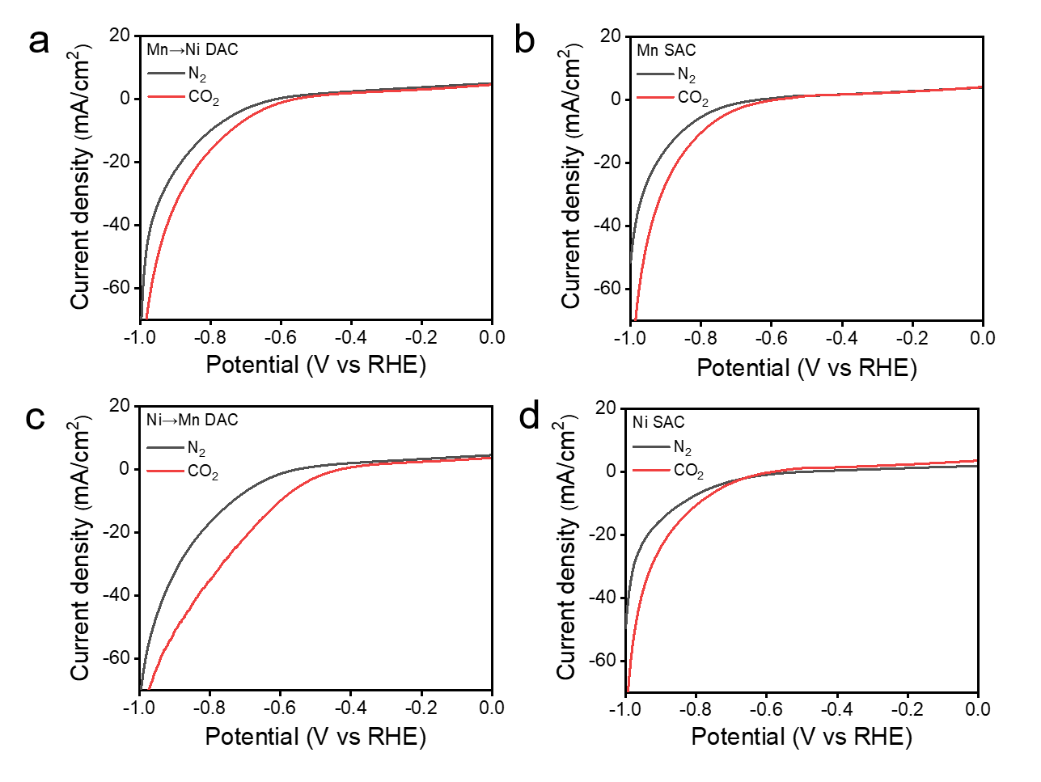


**Figure S6.** LSV curves of all catalysts in N_2_- and CO_2_-saturated 0.5 M KHCO_3_.


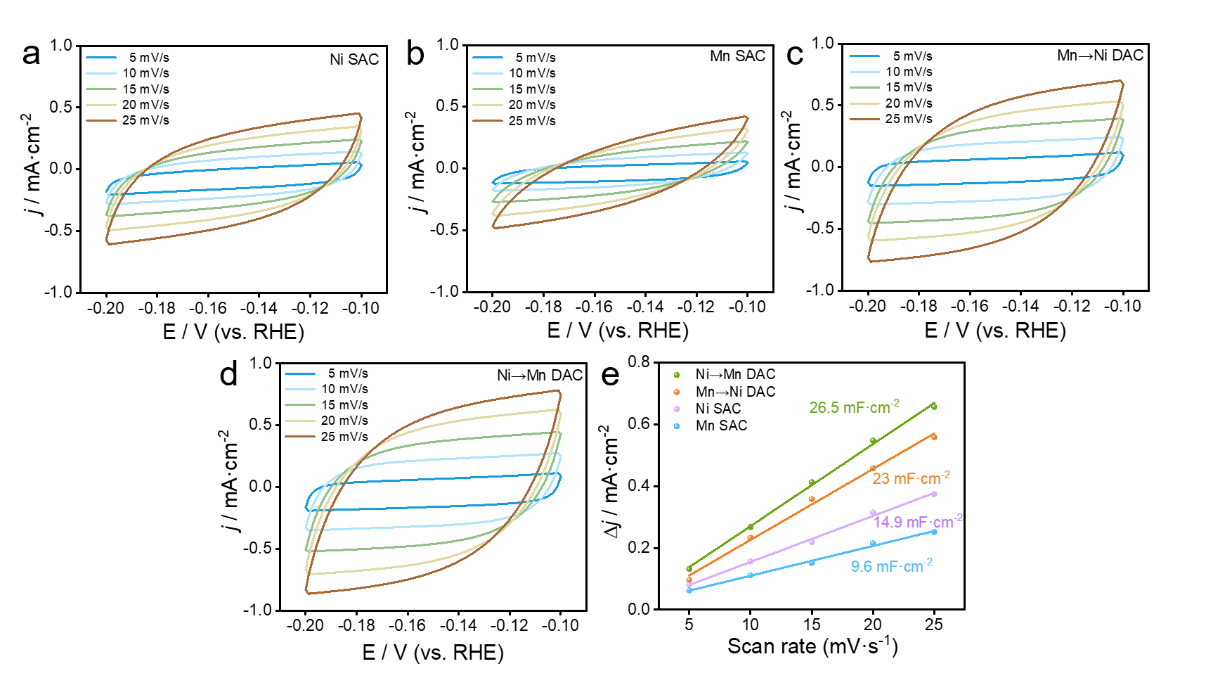


**Figure S7.** CV curves of (a) Ni SAC, (b) Mn SAC, (c) Mn→Ni DAC and (d) Ni→Mn DAC at various scan rates. (e) Evaluation of C_dl_ values by plotting the Δ*j* vs. scan rate.


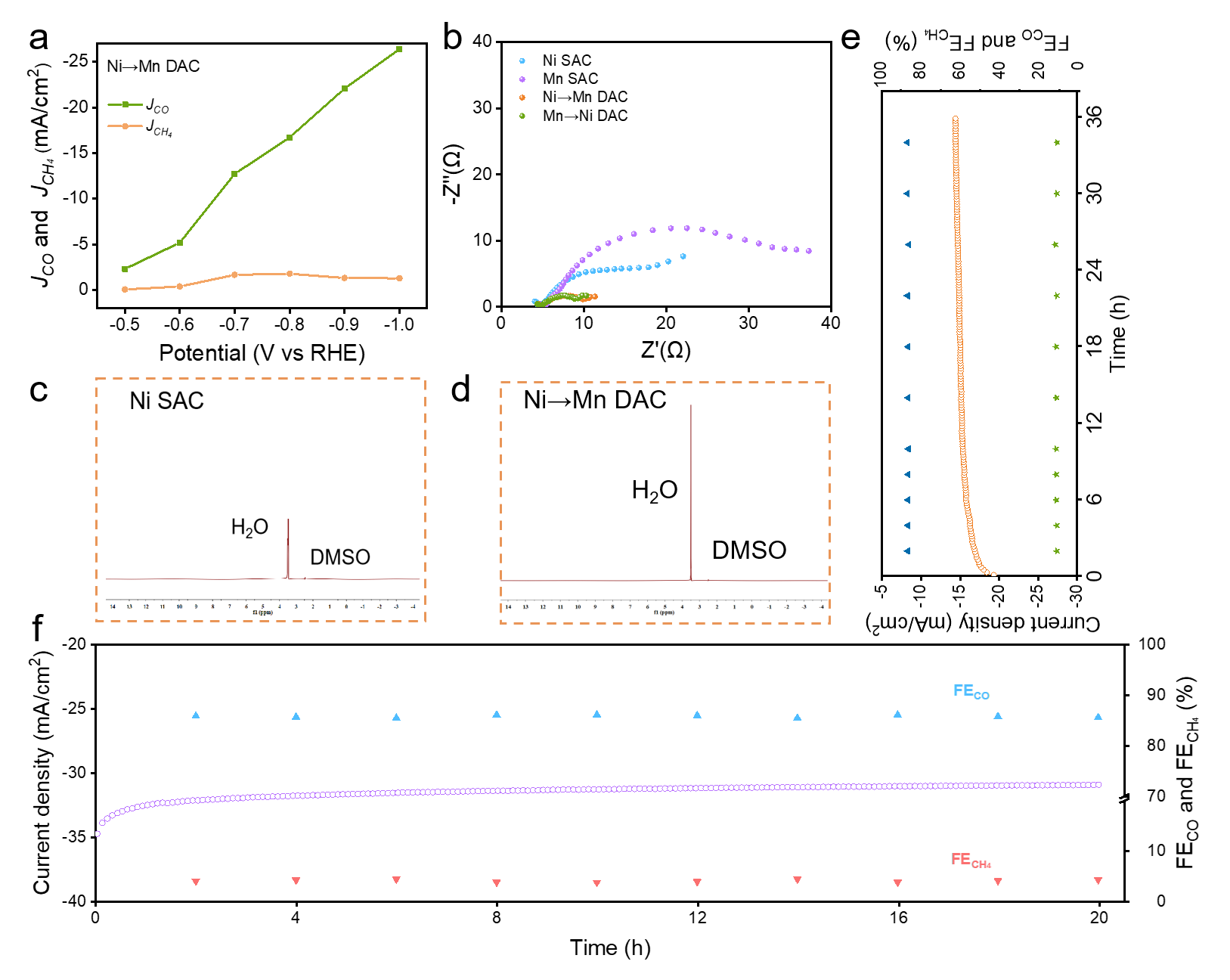


**Figure S8.** (a) The *J*_CO_ and *J*_CH4_ for Ni→Mn DAC in 0.5 M KHCO_3_. (b) The EIS spectra for all catalysts. ^1^H NMR spectra for the electrolyte after electrolysis at the best potential of (c) Ni SAC and (d) Mn SAC. No liquid products were detected in the ^1^H NMR spectra. (e) The long-term cyclic stability of Ni→Mn DAC at -0.7 V (vs RHE). (f) The long-term cyclic stability of Ni→Mn DAC at -1.0 V (vs RHE) in 20-hour intervals.


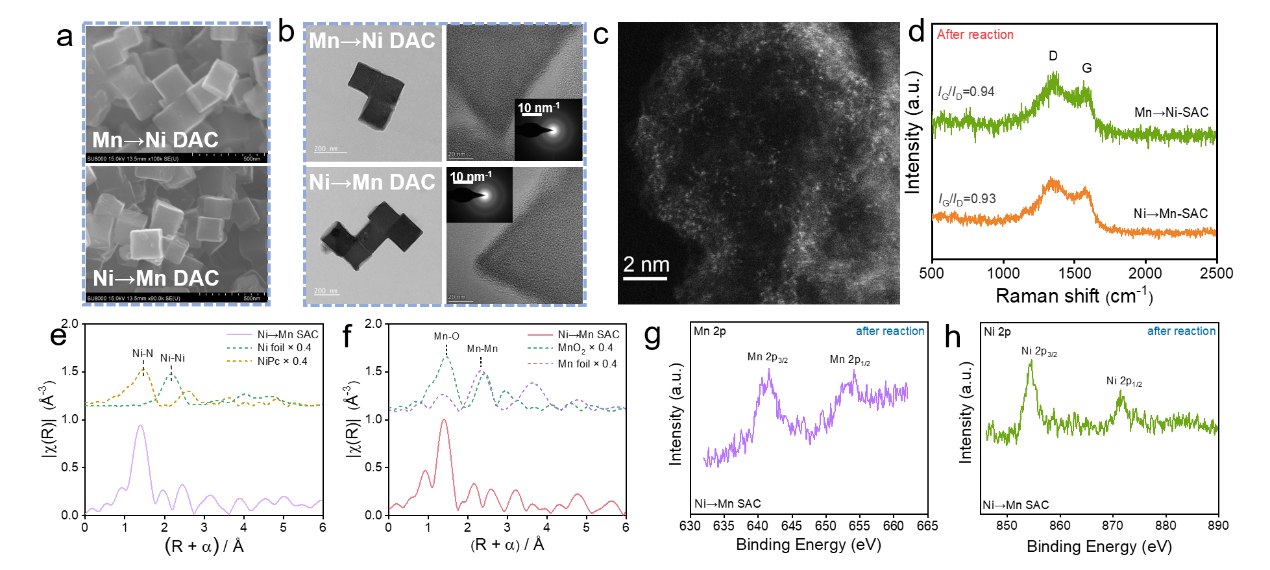


**Figure S9.** (a) SEM images and (b) TEM, HRTEM and SAED images of Mn→Ni DAC and Ni→Mn DAC after long-term electrolysis. (c) HAADF-STEM image after the reaction. (d) The Raman spectra for Mn→Ni DAC and Ni→Mn DAC after long-term electrolysis. FT *k*^2^-weighted (e) Ni *K*-edge and (f) Mn *K*-edge of the EXAFS spectra after the reaction. Post-test XPS of (g) Mn and (h) Ni 2*p* for Ni→Mn DAC.


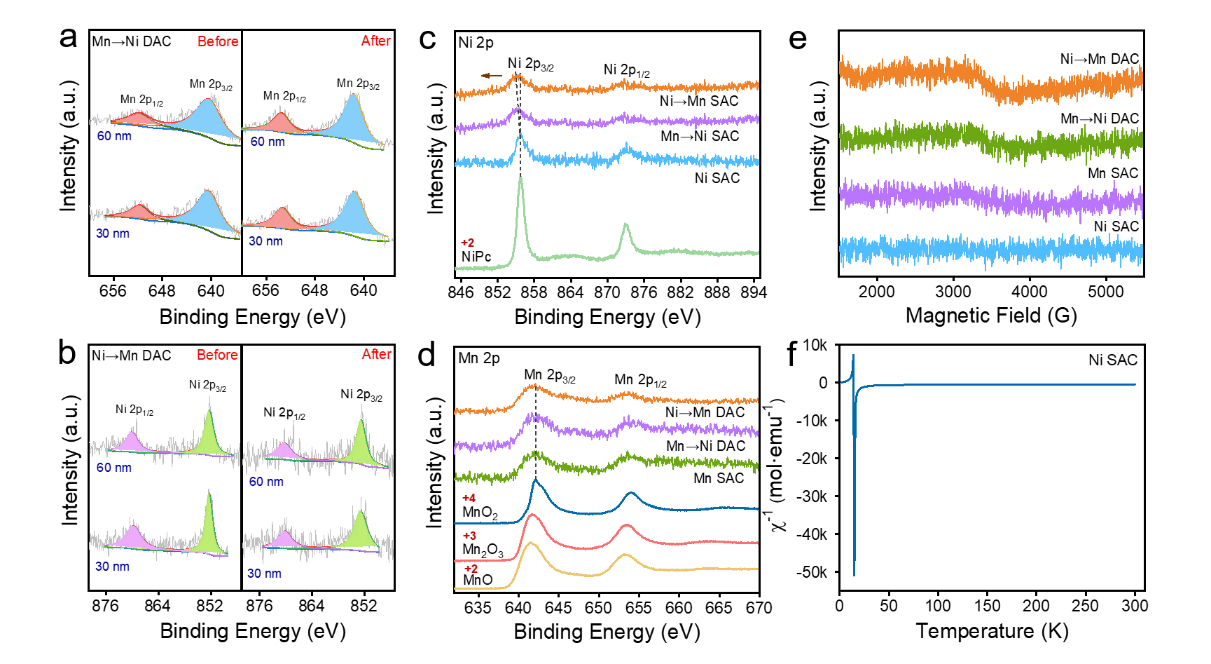


**Figure S10.** (a) High-resolution XPS of Mn 2*p* spectra for Mn→Ni DAC, Ni 2*p* spectra of Ni→Mn DAC with Ar ion sputtering depths of 30 and 60 nm before and after reaction. (b) The Ni 2*p* spectra and Mn 2*p* spectra of the Ni-related, Mn-related catalysts and standard sample before and after reaction. (c) The EPR spectra for all materials. (d) 1/χ plots of Ni SAC.


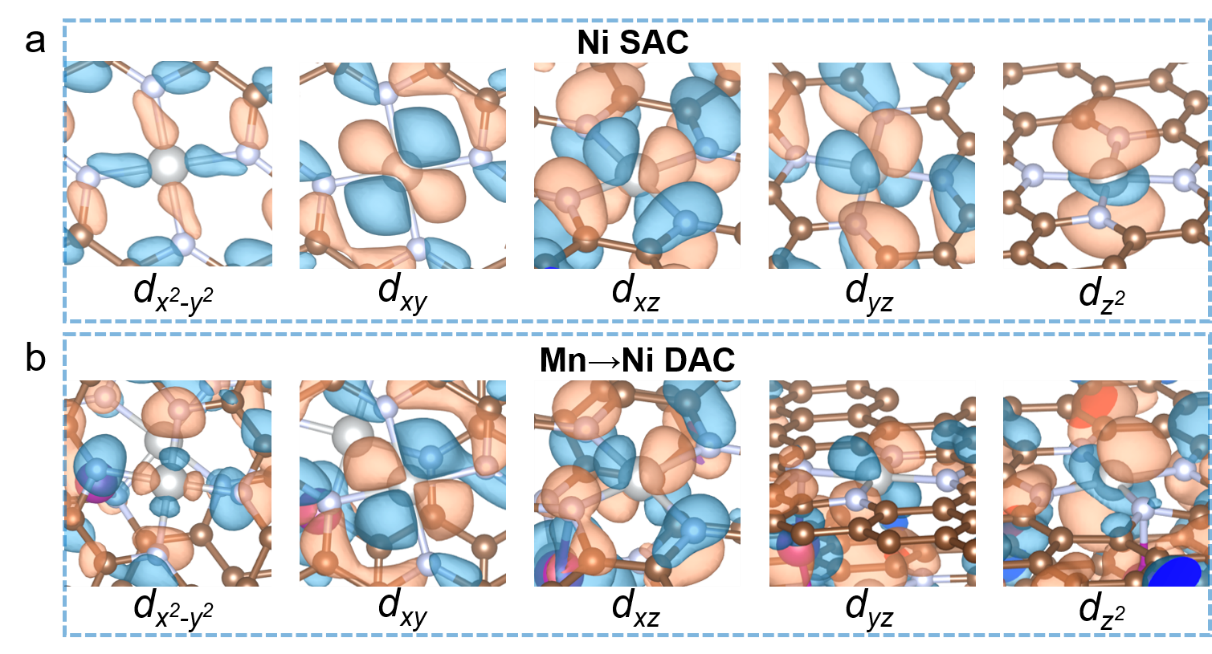


**Figure S11.** The wave functions of Ni 3*d* orbitals with corresponding energy level labels for (a) Ni SAC and (b) Mn→Ni DAC.

**Figure S12.** (a) The corresponding schematic illustration of the free energy diagram for the key intermediates in CH_4_ formation for Mn SAC, Ni→Mn DAC and Mn→Ni DAC. (b) The free energy diagram for the key intermediates of the CH_4_ formation pathway.

**Figure S13.** Charge density difference of (a) Ni→Mn DAC and (b) Mn→Ni DAC.

**Table S1.** Curvefit Parameters for Ni *K*-edge EXAFS (CN: coordination number; R: distance; σ^2^: mean-square disorder; S0^2^ was fixed as 0.8).

| Sample | Path | CN | R/Å | σ^2^/Å |
| --- | --- | --- | --- | --- |
| Ni SAC | Ni-N | 3.7 | 1.86 | 0.004 |
| Mn→Ni DAC | Ni-N | 3.8 | 1.85 | 0.002 |
| Ni→Mn DAC | Ni-N | 1.2 | 1.87 | 0.005 |
|  | Ni-C | 1.1 | 1.75 | 0.003 |

**Table S2.** Curvefit Parameters for Mn *K*-edge EXAFS (S0^2^ was fixed as 0.8).

| Sample | Path | CN | R/Å | σ^2^/Å |
| --- | --- | --- | --- | --- |
| Mn SAC | Mn-N | 3.8 | 1.87 | 0.001 |
| Mn→Ni DAC |  | 1.8 | 1.83 | 0.002 |
| Ni→Mn DAC |  | 3.8 | 1.86 | 0.004 |

**Table S3.** Elemental composition of the catalysts measured by ICP-OES.

| Samples | Ni (wt %) | Mn (wt %) |
| --- | --- | --- |
| Ni SAC | 1.05 | / |
| Mn SAC | / | 1.16 |
| Ni→Mn DAC | 1.13 | 1.21 |
| Mn→Ni DAC | 1.07 | 1.18 |

**Table S4.** Performance comparison of various CO_2_RR catalysts.

| Samples | Applied potential (vs RHE) | *j*_CO_  (-mA cm^-2^) | FE_CO_ | FE_CH4_ | Electrolyte | Ref |
| --- | --- | --- | --- | --- | --- | --- |
| Fe/NG-750 | -0.6 | 1.6 | 80 | <5% | 0.1 M KHCO_3_ | [S1] |
| Co_1_-N_4_ | -0.8 | 15.8 | 82 | 0 | 0.1 M KHCO_3_ | [S2] |
| Ni SAs/N-C | -0.9 | 10.48 | 71.9 | 0 | 0.5 M KHCO_3_ | [S3] |
| Ni-N-C | -0.67 | 3.9 | 93 | 0 | 0.5 M KHCO_3_ | [S4] |
| CuFe-N-C | -0.5 | 2.26 | 95.5 | 0 | 0.5 M KHCO_3_ | [S5] |
| Fe/Cu-N-C | -0.8 | 12.91 | 99.2 | 0 | 0.1 M KHCO_3_ | [S6] |
| Ni/Fe-N-C | -0.7 | 7.4 | 98 | 0 | 0.5 M KHCO_3_ | [S7] |
| Ni→Mn DAC | -0.7 | 12.7 | 86.6 | 11.4 | 0.5 M KHCO_3_ | This work |

**References**

[S1] C. H. Zhang, S. Z. Yang, J. J. Wu, M. J. Liu, S. Yazdi, M. Q. Ren, J. W. Sha, J. Zhong, K. Q. Nie, A. S. Jalilov, Z. Y. Li, H. M. Li, B. I. Yakobson, Q. Wu, E. L. Ringe, H. Xu, P. M. Ajayan, J. M. Tour. Electrochemical CO_2_ Reduction with Atomic Iron-Dispersed on Nitrogen-Doped Graphene. *Adv. Energy Mater.* **2018**, *8*, 1703487.

[S2] Z. G. Geng, Y. J. Cao, W. X. Chen, X. D. Kong, Y. Liu, T. Yao, Y. Lin. Regulating the coordination environment of Co single atoms for achieving efficient electrocatalytic activity in CO_2_ reduction. *Appl. Catal. B-Environ.* **2019**, *240*, 234-240.

[S3] C. M. Zhao, X. Y. Dai, T. Yao, W. X. Chen, X. Q. Wang, J. Wang, J. Yang, S. Q. Wei, Y. E. Wu, Y. D. Li. Ionic Exchange of Metal-Organic Frameworks to Access Single Nickel Sites for Efficient Electroreduction of CO_2_. *J. Am. Chem. Soc.* **2017**, *139*, 8078-8081.

[S4] X. M. Hu, H. H. Hval, E. T. Bjerglund, K. J. Dalgaard, M. R. Madsen, M. M. Pohl, E. Welter, P. Lamagni, K. B. Buhl, M. Bremholm, M. Beller, S. U. Pedersen, T. Skrydstrup, K. Daasbjerg. Selective CO_2_ Reduction to CO in Water using Earth-Abundant Metal and Nitrogen-Doped Carbon Electrocatalysts. *ACS Catal.* **2018**, *8*, 6255-6264.

[S5] F. H. Wang, H. P. Xie, T. Liu, Y. F. Wu, B. Chen. Highly dispersed CuFe-nitrogen active sites electrode for synergistic electrochemical CO_2_ reduction at low overpotential. *Appl. Energy* **2020**, *269*, 115029.

[S6] M. M. Feng, X. M. Wu, H. Y. Cheng, Z. H. Fan, X. C. Li, F. J. Cui, S. Fan, Y. Dai, G. P. Lei, G. H. He. Well-defined Fe-Cu diatomic sites for efficient catalysis of CO_2_ electroreduction. *J. Mater. Chem. A.* **2021**, *9*, 23817-23827.

[S7] W. H. Ren, X. Tan, W. F. Yang, C. Jia, S. M. Xu, K. X. Wang, S. C. Smith, C. Zhao. Isolated Diatomic Ni-Fe Metal-Nitrogen Sites for Synergistic Electroreduction of CO_2_. *Angew. Chem., Int. Edit.* **2019**, *58*, 6972-6976.
